# Phylogenetic analysis and antigenic epitope prediction for E6 and E7 of Alpha-papillomavirus 9 in Taizhou, China

**DOI:** 10.1186/s12864-024-10411-1

**Published:** 2024-05-22

**Authors:** Haobo Yuan, Ziyi Yan, Jun Gan, Xinghong Di, Yi Qiu, Huihui Xu

**Affiliations:** 1 School of Medicine, Shaoxing University, Shaoxing, Zhejiang 312000 P. R. China; 2 Medical Research Center, Taizhou Hospital of Zhejiang Province, Wenzhou Medical University, Linhai, Zhejiang 317000 P. R. China; 3 Key Laboratory of Minimally Invasive Techniques & Rapid Rehabilitation of Digestive System Tumor of Zhejiang Province, Linhai, Zhejiang 317000 P. R. China; 4 Scientific Research Department, Taizhou Hospital of Zhejiang Province, Wenzhou Medical University, Linhai, Zhejiang 317000 P. R. China

**Keywords:** Human papillomavirus, Alpha-papillomavirus 9, Genetic variations, Phylogenetics, Epitope prediction, Immunoinformatics

## Abstract

**Background:**

Alpha-papillomavirus 9 (α-9) is a member of the human papillomavirus (HPV) α genus, causing 75% invasive cervical cancers worldwide. The purpose of this study was to provide data for effective treatment of HPV-induced cervical lesions in Taizhou by analysing the genetic variation and antigenic epitopes of α-9 HPV E6 and E7.

**Methods:**

Cervical exfoliated cells were collected for HPV genotyping. Positive samples of the α-9 HPV single type were selected for *E6* and *E7* gene sequencing. The obtained nucleotide sequences were translated into amino acid sequences (protein primary structure) using MEGA X, and positive selection sites of the amino acid sequences were evaluated using PAML. The secondary and tertiary structures of the E6 and E7 proteins were predicted using PSIPred, SWISS-MODEL, and PyMol. Potential T/B-cell epitopes were predicted by Industrial Engineering Database (IEDB).

**Results:**

From 2012 to 2023, α-9 HPV accounted for 75.0% (7815/10423) of high-risk HPV-positive samples in Taizhou, both alone and in combination with other types. Among these, single-type-positive samples of α-9 HPV were selected, and the entire *E6* and *E7* genes were sequenced, including 298 HPV16, 149 HPV31, 185 HPV33, 123 HPV35, 325 HPV52, and 199 HPV58 samples. Compared with reference sequences, 34, 12, 10, 2, 17, and 17 nonsynonymous nucleotide mutations were detected in HPV16, 31, 33, 35, 52, and 58, respectively. Among all nonsynonymous nucleotide mutations, 19 positive selection sites were selected, which may have evolutionary significance in rendering α-9 HPV adaptive to its environment. Immunoinformatics predicted 57 potential linear and 59 conformational B-cell epitopes, many of which are also predicted as CTL epitopes.

**Conclusion:**

The present study provides almost comprehensive data on the genetic variations, phylogenetics, positive selection sites, and antigenic epitopes of α-9 HPV E6 and E7 in Taizhou, China, which will be helpful for local HPV therapeutic vaccine development.

**Supplementary Information:**

The online version contains supplementary material available at 10.1186/s12864-024-10411-1.

## Background

Cervical cancer remains the fourth most common cancer affecting women’s health worldwide, especially in China. Persistent high-risk HPV infection is considered the primary aetiological factor for cervical cancer [1]. HPV is a small, double-stranded, circular DNA virus that exclusively infects epithelial cells of the skin or mucosa. The genome size of HPV is approximately 8.0 kb, containing six early genes (*E1, E2, E4, E5, E6, E7*), two late genes (*L1, L2*), and long control region (LCR). The oncoproteins encoded by the *E6* and *E7* genes play a crucial role in HPV-driven viral carcinogenesis and cancers [2, 3]. E6 binds to the tumour suppressor protein p53 and prevents its translocation, and mediate the cellular transformation by inhibiting the ability of p53 to activate. E7 binds to the retinoblastoma protein (Rb) and induces cells to enter into premature S-phase by disrupting Rb-E2F complexes. These processes lead to impaired p53 and Rb functions, involving DNA repair, cell cycle, apoptosis, and ultimately result in immortalization of HPV-infected cells [4].

To date, over 200 HPVs have been identified, which are divided into five main genera referred to as alpha (α)-, beta (β)-, gamma (γ)-, Mu(μ)-, and Nu(ν)-papillomaviruses [5]. In addition, these HPVs are divided into high- and low-risk types based on their carcinogenic potential. Of which, at least 14 are classified as high-risk HPV types, such as HPV16, 18, 31, 33, 35, 39, 45, 51, 52, 56, 58, 59, 66 and 68. It is worth noting that almost all high-risk HPV types belong to the α genus, including α-5 (HPV51), α-6 (HPV56, 66), α-7 (HPV18, 39, 45, 59, 68), and α-9 (HPV16, 31, 33, 35, 52, 58). Of which, α-9 species causes approximately 75% invasive cervical cancers worldwide [6]. Our previous study suggested that women infected with HPV16, 18, 31, 33, 52, or 58 are more likely to have CIN2 + lesions [7]. Moreover, the incidence of CIN2 + caused by HPV33, 52, and 58 was greater than that caused by HPV 18 in Taizhou [7]. Different HPV types exhibit differences in their immunogenicity, adaptability, and carcinogenicity, which may be due to different genetic variations and selection pressures during the evolutionary process [8, 9]. Therefore, focus should be placed on these common carcinogenic HPV types, such as α-9 species in the Taizhou region.

Persistent infection of human epithelial cells by HPV leads to integration of the viral DNA into the host genome, usually disrupting the E1 and/or E2 genes [10]. HPV integration is a key event for cervical carcinogenesis, leading to structural aberrations in the host genome or abnormal gene expression of target genes. Of which, the expression of E6 and E7 oncoproteins cause cellular immortalization and neoplastic transformation. The most frequent integration sites include SHKBP1, ERBB3, CASP8, HLA-A, HLA-B, TGFBR2, PIK3CA, EP300, FBXW7, PTEN, NFE2L2, ARID1A, KRAS, MAPK1, etc. [11, 12]. The transformed cervical cells show expression of HPV E6 or E7, with antigenic epitopes on their protein surface that stimulate B lymphocytes to produce antibodies [13, 14]. Viral antigen peptides are presented at the cell surface through human leukocyte antigen (HLA) and are recognized by CD8 + cytotoxic T lymphocytes (CTLs) [15–17]. Therefore, E6 or E7 molecules are considered ideal targets for HPV therapeutic vaccines, inducing cell-mediated immunity by stimulating CTLs in immune response strategies or eliciting humoral immunity by activating B lymphocytes to produce specific antibodies [18, 19].

Unfortunately, as a major issue for cervical cancer, local data available on HPV therapeutic vaccines are still limited in China, and almost no consideration is given to *E6* or *E7* gene mutations. Hence, there is an urgent need to further study the genetic variation, positive selection site, protein structure, and antigen epitopes of α-9 HPV E6 or E7 to provide data to explore effective treatment of HPV-induced cervical lesions in Taizhou, China.

## Materials and methods

### Study population

This study was ethically approved by the Institutional Medical Ethics Review Board of Taizhou Hospital, China. Cervical exfoliated cells were collected from women who underwent cervical cancer screening at Taizhou Hospital. The specimens were collected by cervical scraping and stored in 2.5 ml of cell preservation buffer at -20 °C. Before specimen collection, written informed consent was obtained from all participants, and the participants’ privacy was strictly protected.

### HPV genotyping

HPV types were identified using the HPV Genotyping Kit (Tellgen Corporation, China), which was approved by the China’s FDA (Certified No. (2014): 3,400,847). The protocol for HPV genotyping has been described in detail in our previous studies [7, 20]. Briefly, the kit uses a set of biotinylated amplimers and multiplex HPV genotyping methods with bead-based Luminex suspension array technology, which is able to simultaneously identify 14 high-risk HPV types including 16, 18, 31, 33, 35, 39, 45, 51, 52, 56, 58, 59, 66, 68 and β-globin gene (internal control).

### PCR amplification and sequencing

Based on the reference sequences of α-9 HPV types in GenBank, specific primer pairs for the entirety of the *E6* and *E7* regions were designed using the Primer-BLAST tool (ncbi.nlm.nih.gov/tools/primer-blast). The primers, PCR conditions, amplicon size, and reference sequences are listed in Table S1. Genomic DNA was extracted using a DNA Extraction Kit (#GK0122, GENEray, China). PCR products were purified and sequenced at BGI, and all data were confirmed by repeating PCR and sequencing reactions at least twice. In this study, genetic variant data for HPV31 and 35 were combined with data from our previous studies on HPV16, 33, 52 and 58 [21–24].

### Phylogenetic analysis and homology comparison

All successfully acquired nucleotide sequences were aligned by BioEdit. Then, a phylogenetic tree of α-9 HPV *E6* and *E7* variation patterns was constructed by the maximum-likelihood method with one thousand bootstrap replicates using MEGA X. The phylogenetic tree was constructed using 201 complete α-9 HPV *E6* and *E7* sequences, including 64 HPV16, 16 HPV31, 15 HPV33, 5 HPV35, 27 HPV52, 25 HPV58, and 49 reference sequences downloaded from NCBI.

### Selective pressure analysis

The CodeML program in PAML (abacus.gene.ucl.ac.uk/software/paml.html) was used to calculate the nonsynonymous (dN)/synonymous (dS) ratio (ω) for selective pressure analysis. If nonsynonymous mutations are favoured by Darwinian selection, they will be fixed at a higher rate than synonymous mutations, resulting in dN > dS, ω = dN/dS > 1 [25].

### Protein structure analysis

The obtained nucleotide sequences were translated into amino acid sequences (protein primary structure) using MEGA X. Then, the secondary and tertiary structures of the E6 and E7 proteins of α-9 HPV were predicted using PSIPred (bioinf.cs.ucl.ac.uk/psipred) and Swiss-model (swissmodel.ExPASy.org), respectively.

### Prediction of linear and conformational B-cell epitopes

Linear B-cell epitopes were predicted from the primary sequence of the E6 or E7 protein using sequence-based methods (Kolaskar and Tongaonkar’s antigenicity, tools.immuneepitope.org/bcell/). Conformational B-cell epitopes were predicted from the tertiary structure of E6 or E7 using ElliPro, which identifies protrusions in antigen surfaces (tools.immuneepitope.org/ellipro).

### Prediction of cytotoxic T-cell epitopes

CTL epitopes were predicted from the E6 or E7 protein of α-9 HPV types using the NetCTL server, which accepts the FASTA format (tools.immuneepitope.org/netchop). NetCTL gives results for 9-mer peptides together with their predicted MHC binding affinity, binding affinity rescale value, C-terminal cleavage affinity, and TAP transport efficiency. C-terminal cleavage weights and TAP transport efficiency were calculated using the default values 0.15 and 0.05, respectively; 9-mer peptides with a prediction score > 0.75 were considered to be potential CTL epitopes.

## Results

### The prevalence of α-9 HPV types in Taizhou

**Fig. 1:**
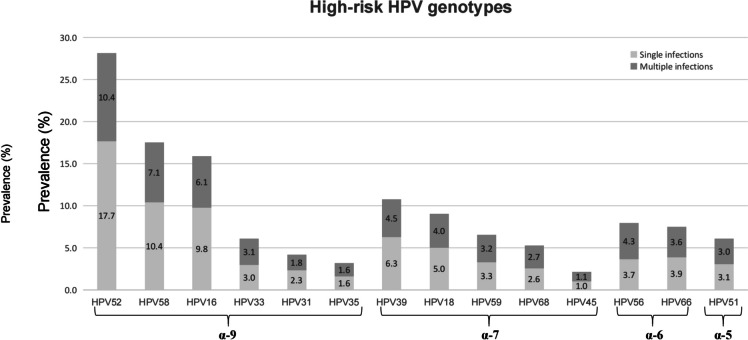
Distribution of different high-risk HPV genotypes in Taizhou from 2012 to 2023

A total of 60,259 women underwent the first round of HPV screening in Taizhou Hospital from December 2012 to June 2023. The overall rate of high-risk HPV infection was 17.3% (10,423/60,259), including infection with multiple HPV types (4.5%). All high-risk HPV-positive samples belonged to the α genus, including α-5 (782, 7.5%), α-6 (1466, 14.1%), α-7 (3521, 33.8%), and α-9 (7815, 75.0%) (Fig. 1, Table S2). α-9 HPV accounted for 75.0% of the high-risk HPV-positive samples in Taizhou, both alone and in combination with other types. In this study, we selected the α-9 HPV single-type-positive samples (HPV 16, 31, 33, 35, 52, 58) for subsequent analyses, including 298 HPV16, 149 HPV31, 185 HPV33, 123 HPV35, 325 HPV52, and 199 HPV58 samples. The clinicopathological information of the study population are shown in Table S3.

### Nucleotide variations of α-9 HPV types

Compared with the reference sequences from GenBank, 53 nucleotide mutations were detected in HPV16, with 34 (34/53, 64.2%) nonsynonymous mutations (24 in *E6* and 10 in *E7*). In addition, 12 (12/24, 50.0%), 10 (10/20, 50.0%), 2 (2/7, 28.6%), 17 (17/35, 48.6%), and 17 (17/26, 65.4%) nonsynonymous nucleotide mutations were detected in the HPV31, 33, 35, 52, and 58 *E6* and *E7* genes, respectively. A summary of nucleotide variations and amino acid substitutions in the E6 and E7 regions of α-9 HPV are shown in Tables 1 and 2.

**Table 1:** Nucleotide variations and amino acid substitutions in α-9 HPV E6 region

HPV16(*n* = 298)																			
No	1	2	3	4	5	6	7	8	9	10	11	12	13	14	15	16	17	18	19
Position	94	95	100	105	110	126	131	134	137	142	145	152	168	176	178	178	184	185	188
Ref K02718	G	A	T	T	C	G	A	A	T	A	G	A	C	G	T	T	A	T	G
Mutatiion	A	G	C	G	G	A	C	C	G	G	T	G	G	A	G	A	G	G	C
Frequency	11	4	1	2	1	1	8	1	1	1	1	1	1	2	192	7	1	2	1
% of Samples	3.7	1.3	0.3	0.7	0.3	0.3	2.7	0.3	0.3	0.3	0.3	0.3	0.3	0.7	64.4	2.3	0.3	0.7	0.3
aa mutation	-	R5G	-	M8R	Q10E	R15Q	-	K18Q	L19V	-	Q21H	T24A	T29S	D32N	D32E	-	I34M	L35V	E36Q
Secondary structure	C	C	C	C	C	C	C	C	H	H	H	H	C	H	H	H	E	E	E
HPV31(*n* = 149)																			
No	1	2	3	4	5	6	7	8	9	10	11	12	13	14	15	16			
Position	134	176	261	285	297	299	320	320	326	404	407	428	446	475	520	537			
Ref J04353	T	C	A	C	A	A	A	A	A	G	A	A	A	A	C	C			
Mutatiion	A	T	C	T	G	G	G	T	G	A	G	G	C	G	T	G			
Frequency	4	46	3	60	3	1	1	63	39	60	1	60	1	3	63	2			
% of Samples	0.03	0.3	0.02	0.4	0.02	0.01	0.01	0.4	0.3	0.4	0.007	0.4	0.007	0.02	0.4	0.01			
aa mutation	-	-	I52L	H60Y	T64A	T64A	-	-	-	-	-	-	E113D	K123R	I138V	P143G			
Secondary structure	C	H	E	E	H	H	H	H	H	C	E	C	H	C	C	C			
HPV33(*n* = 185)																			
No	1	2	3	4	5	6	7	8	9	10	11	12	13	14					
Position	177	196	213	245	273	315	364	387	446	447	458	480	531	549					
Ref M12732	A	T	A	C	A	C	A	A	A	A	G	A	G	T					
Mutatiion	T	G	C	T	G	T	C	C	G	T	T	T	A	C					
Frequency	2	1	48	1	43	4	45	45	43	1	1	2	1	3					
% of Samples	1.1	0.5	25.9	0.5	23.2	2.2	24.3	24.3	23.2	0.5	0.5	1.1	0.5	1.6					
aa mutation	-	C30G	K35N	A46V	-	-	N86H	K93N	Q113R	-	R117L	-	-	-					
Secondary structure	C	C	C	H	E	H	H	C	H	H	H	C	C	C					
HPV35(*N* = 123)																			
No	1	2	3	4	5														
Position	127	136	274	341	370														
Ref HQ537708	T	T	A	T	G														
Mutatiion	C	C	G	C	C														
Frequency	122	122	3	122	40														
% of Samples	0.99	0.99	0.02	0.99	0.33														
aa mutation	-	-	-	W78R	-														
Secondary structure	C	C	E	C	H														
HPV52(*n* = 325)																			
No	1	2	3	4	5	6	7	8	9	10	11	12	13	14	15	16	17	18	
Position	108	180	191	237	267	292	309	317	329	338	347	350	356	378	378	379	467	530	
Ref HQ537732	G	A	T	C	G	T	T	G	T	T	A	G	G	A	A	A	C	A	
Mutatiion	C	C	C	T	A	G	C	A	C	C	G	T	A	G	C	G	A	G	
Frequency	1	1	1	5	1	1	1	1	1	1	2	321	73	8	7	313	4	7	
% of Samples	0.3	0.3	0.3	1.5	0.3	0.3	0.3	0.3	0.3	0.3	0.6	98.8	22.5	2.5	2.2	96.3	1.2	2.2	
aa mutation	E3Q	-	-	-	D56N	I64S	-	-	-	-	-	-	-	K93G	K93Q	K93R	N122K	-	
Secondary structure	C	E	C	H	C	H	H	H	H	C	E	E	H	C	C	C	C	C	
HPV58(*n* = 199)																			
No	1	2	3	4	5	6	7	8	9	10	11	12	13	14					
Position	145	152	187	194	217	225	228	259	260	307	388	390	432	534					
Ref NC001443	G	T	C	A	T	G	C	A	A	C	A	A	G	C					
Mutatiion	A	C	T	C	A	A	T	G	C	T	C	C	A	A					
Frequency	1	1	1	1	1	2	1	1	1	64	87	1	1	1					
% of Samples	0.5	0.5	0.5	0.5	0.5	1.0	0.5	0.5	0.5	32.2	43.7	0.5	0.5	0.5					
aa mutation	-	-	-	K29Q	-	R39Q	S40F	-	-	-	K93N	K94T	R108K	P142H					
Secondary structure	H	H	E	E	C	H	H	C	E	H	C	C	C	C					

HPV16(*n* = 298)																		
No	20	21	22	23	24	25	26	27	28	29	30	31	32	33	34	35	36	37
Position	193	219	267	276	278	296	310	310	335	350	373	412	430	434	442	452	473	505
Ref K02718	T	G	G	A	C	A	T	T	C	T	G	T	A	T	A	A	A	T
Mutatiion	C	A	A	G	T	C	G	C	T	G	A	C	T	G	C	C	C	G
Frequency	1	1	1	2	1	1	1	2	24	13	1	1	1	1	34	1	2	1
% of Samples	0.3	0.3	0.3	0.7	0.3	0.3	0.3	0.7	8.1	4.4	0.3	0.3	0.3	0.3	11.4	0.3	0.7	0.3
aa mutation	-	R46Q	R62K	N65S	P66S	K72Q	F76L	-	H85Y	L90V	-	-	-	C118G	E120D	-	-	-
Secondary structure	C	H	E	E	E	H	H	H	C	E	H	C	C	C	H	H	C	C
HPV31(*n* = 149)																		
No																		
Position																		
Ref J04353																		
Mutatiion																		
Frequency																		
% of Samples																		
aa mutation																		
Secondary structure																		
HPV33(*n* = 185)																		
No																		
Position																		
Ref M12732																		
Mutatiion																		
Frequency																		
% of Samples																		
aa mutation																		
Secondary structure																		
HPV35(*N* = 123)																		
No																		
Position																		
Ref HQ537708																		
Mutatiion																		
Frequency																		
% of Samples																		
aa mutation																		
Secondary structure																		
HPV52(*n* = 325)																		
No																		
Position																		
Ref HQ537732																		
Mutatiion																		
Frequency																		
% of Samples																		
aa mutation																		
Secondary structure																		
HPV58(*n* = 199)																		
No																		
Position																		
Ref NC001443																		
Mutatiion																		
Frequency																		
% of Samples																		
aa mutation																		
Secondary structure																		

Predicted amino acid changes were also shown in the last low. The "C" means coil, “E” means β-Strand, the “H” means α-Helix

**Table 2:** Nucleotide variations and amino acid substitutions in α-9 HPV E7 region

HPV16(*n* = 298)																	
No	1	2	3	4	5	6	7	8	9	10	11	12	13	14	15	16	
Position	619	627	646	647	647	663	676	712	730	760	789	790	791	823	843	846	
Ref K02718	A	C	A	A	A	G	G	C	T	T	T	C	G	G	T	T	
Mutatiion	T	T	C	G	C	A	C	A	C	C	C	T	T	T	C	C	
Frequency	1	1	28	195	1	40	1	1	1	7	1	19	1	1	41	192	
% of Samples	0.3	0.3	9.4	65.4	0.3	13.4	0.3	0.3	0.3	2.3	0.3	6.4	0.3	0.3	13.8	64.4	
aa mutation	T20S	-	N29H	N29S	N29T	-	D39H	H51N	F57L	-	-	R77C	R77L	G88^*^	-	-	
Secondary structure	C	C	C	C	C	C	C	C	E	E	H	H	H	C	C	C	
HPV31(*n* = 149)																	
No	1	2	3	4	5	6	7	8									
Position	580	626	670	695	743	787	788	826									
Ref J04353	G	C	C	G	A	T	C	C									
Mutatiion	A	T	T	A	G	C	T	T									
Frequency	60	86	64	63	149	1	2	2									
% of Samples	0.4	0.6	0.4	0.4	1	0.01	0.01	0.01									
aa mutation	-	H23Y	-	E46K	K62E	-	R77C	-									
Secondary structure	C	C	C	C	C	H	H	E									
HPV33(*n* = 185)																	
No	1	2	3	4	5	6											
Position	704	706	706	737	740	862											
Ref M12732	A	C	C	A	C	A											
Mutatiion	G	T	A	G	T	T											
Frequency	1	5	3	2	1	45											
% of Samples	0.5	2.7	1.6	1.1	0.5	24.3											
aa mutation	-	A45V	A45E	-	-	Q97L											
Secondary structure	C	C	C	E	E	C											
HPV35(*N* = 123)																	
No	1	2															
Position	675	748															
Ref HQ537708	T	G															
Mutatiion	C	A															
Frequency	1	2															
% of Samples	0.01	0.02															
aa mutation	-	E63K															
Secondary structure	C	C															
HPV52(*n* = 325)																	
No	1	2	3	4	5	6	7	8	9	10	11	12	13	14	15	16	17
Position	573	582	595	662	664	706	707	727	733	742	751	801	822	834	843	848	849
Ref HQ537732	T	T	C	C	G	A	G	T	C	G	C	A	A	C	A	T	A
Mutatiion	A	G	T	T	A	G	A	G	T	A	T	G	G	T	G	G	C
Frequency	7	2	2	7	1	7	7	7	7	8	314	321	1	2	2	7	10
% of Samples	2.2	0.6	0.6	2.2	0.3	2.2	2.2	2.2	2.2	2.5	96.6	98.8	0.3	0.6	0.6	2.2	3.1
aa mutation	-	D10E	-	T37I	D38N	S52D	S52D	Y59D	H61Y	D64N	-	-	-	-	-	L99R	L99R
Secondary structure	C	H	C	C	C	C	C	E	C	C	E	H	C	C	H	C	C
HPV58(*n* = 199)																	
No	1	2	3	4	5	6	7	8	9	10	11	12					
Position	599	632	678	694	726	744	755	756	760	761	763	803					
Ref NC001443	G	C	T	G	T	T	C	T	G	G	A	T					
Mutatiion	A	T	A	A	C	G	A	C	A	A	G	C					
Frequency	4	24	1	40	3	121	1	1	24	39	1	57					
% of Samples	2.0	12.1	0.5	20.1	1.5	60.8	0.5	0.5	12.1	19.6	0.5	28.6					
aa mutation	R9K	T20I	D35E	G41R	-	-	T61N	T61N	G63S	G63D	T64A	V77A					
Secondary structure	H	C	C	C	C	E	C	C	C	C	C	H					

Predicted amino acid changes were also shown in the last low. The "C" means coil, “E” means β-Strand, the “H” means α-Helix

Of all genetic variations in our study, 31 newly reported nonsynonymous mutations have never been reported: T105G(M8R), C110G(Q10E), G126A(R15Q), A134C(K18Q), T137G(L19V), A152G(T24A), A296C(K72Q), T310G(F76L), T434G(C118G) in HPV16 E6, and A619T(T20S), A647C(N29T), G676C(D39H), T730C(F57L), G791T(R77L), G823T(G88*) in HPV16 E7; A299G(T64A), A446C(E113D), C537G(P143G) in HPV31 E6, and C788T(R77C) in HPV31 E7; T196G (C30G), G458T (R117L) in HPV33 E6; G108C(E3Q), G267A(D56N), T292G(I64S) in HPV52 E6; A194C(K29Q), G225A(R39Q), C228T(S40F), A390C(K94T), G432A(R108K), and C534A(P142H) in HPV58 E6 and T678A(D35E) in HPV58 E7.

### Phylogenetic construction

**Fig. 2:**
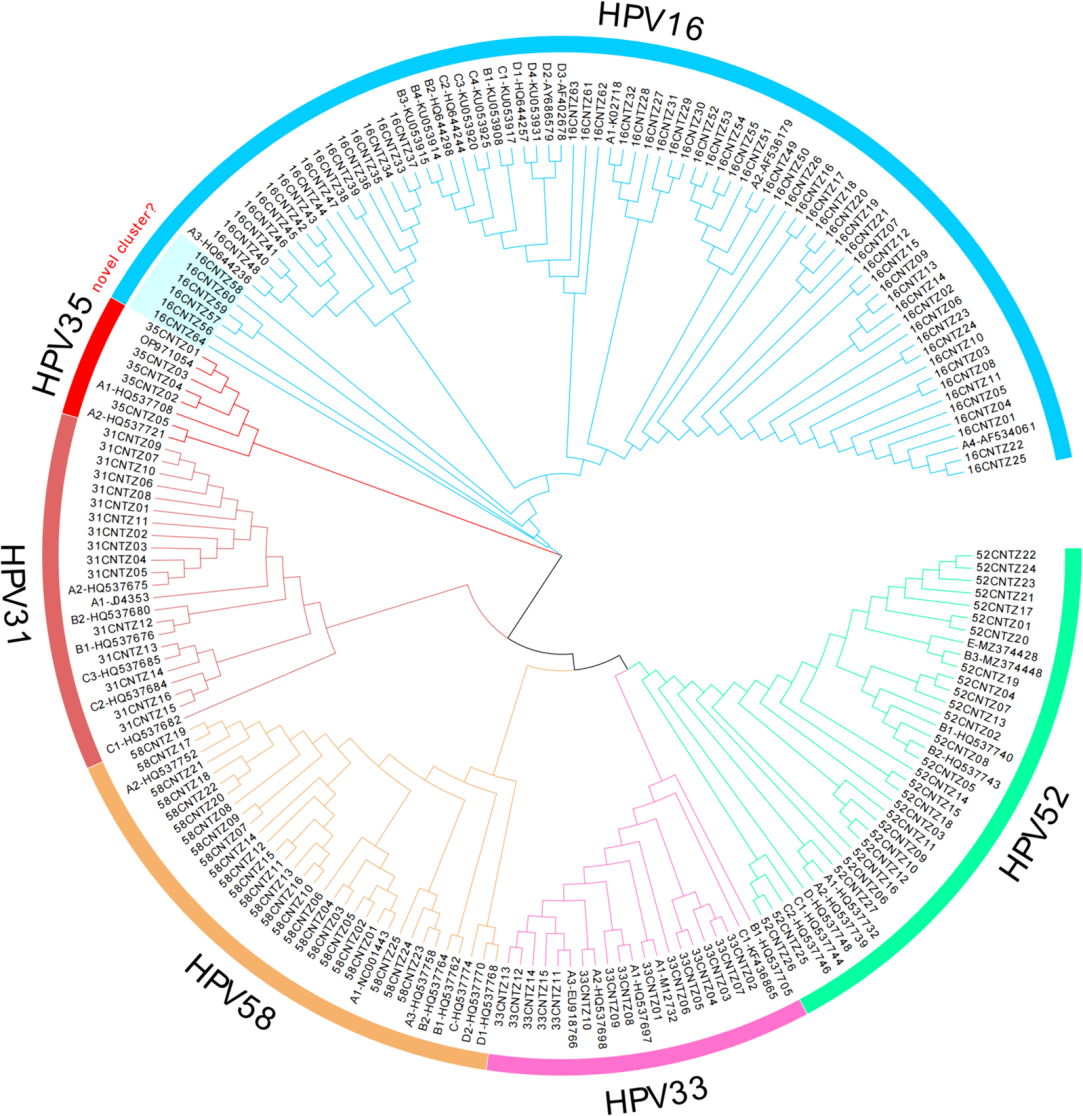
Phylogenetic tree of α-9 HPV variants. Maximum-likelihood analysis (with MEGA X) of *E6* and *E7* nucleotide sequences was inferred from 201 complete α-9 HPV *E6*/*E7* sequences, including 64 HPV16, 16 HPV31, 15 HPV33, 5 HPV35, 27 HPV52, 25 HPV58, and 49 reference sequences

All nucleotide sequences of this study were submitted to GenBank, and accession numbers were obtained (HPV16E6E7: MT681266-MT681329, HPV31E6E7: OR540563-OR540578, HPV33E6E7: OQ672665-OQ672679, HPV35E6E7: OR540579-OR540583, HPV52E6E7: ON529577-ON529603, HPV58E6E7: MH348918-MH348942). A phylogenetic tree was constructed from 201 complete α-9 HPV *E6* and *E7* nucleotide sequences (152 obtained from our study and 49 downloaded from GenBank) (Fig. 2). Furthermore, we constructed phylogenetic trees based on the E6 and E7 amino acid sequences (Figures S1- S2).

### Selective pressure analysis

The results of dN/dS analysis for α-9 HPV *E6* and *E7* genes showed a high nonsynonymous mutation rate for positively selected sites. Nineteen positive selection sites were detected, including 32E of HPV16 E6, 93K of HPV33 E6, 93R of HPV52 E6, 29S*, 77R*, 88-** of HPV16 E7, 45A, 97Q of HPV33 E7, 63E of HPV35 E7, 52S, 64D of HPV52 E7, 9R, 20T, 35D, 41G, 61T, 63G, 64T, and 77V of HPV58 E7. However, no reliable HPV31 E6, HPV35 E6, HPV58 E6, or HPV31 E7 positive selection sites were detected (Table 3).

**Table 3:** Positive section sites of α-9 HPV E6 and E7 sequences

Model	HPV16^a^	HPV31	HPV33	HPV35	HPV52	HPV58	HPV16^b^	HPV31	HPV33	HPV35	HPV52	HPV58
	E6 positive selected sites						E7 positive selected sites					
M7	NA	NA	NA	NA	NA	NA	NA	NA	NA	NA	NA	NA
M8	32E^c^	NA	93 K	NA	93R	NA	29S^c^	NA	45A	63E	52S	9R
							77R^c^		97Q		64D	20 T
							88-^d^					35D
												41G
												61 T
												63G
												64 T
												77 V

When the posterior probability was ≥ 0.9, the BEB method was used to identify the positive selection sites

M7 means NEB (Native Empirical Bayes) model, M8 means BEB (Bayes Empirical Bayes) model, NA means not apply

^a^InL = -966.58, Estimates of parameters, p0 = 0.99127, *p* = 53.85297, q = 99.00, p1 = 0.00873, ω = 8.78194

^b^InL = -651.0457, Estimates of parameters, p0 = 0.9897, *p* = 0.00500, q = 0.01600, p1 = 0.010, ω = 999.00

^c^means a posteriori probability ≥ 0.95

^d^means a posteriori probability ≥ 0.99

### Protein structure analysis and homology modelling

**Fig. 3:**
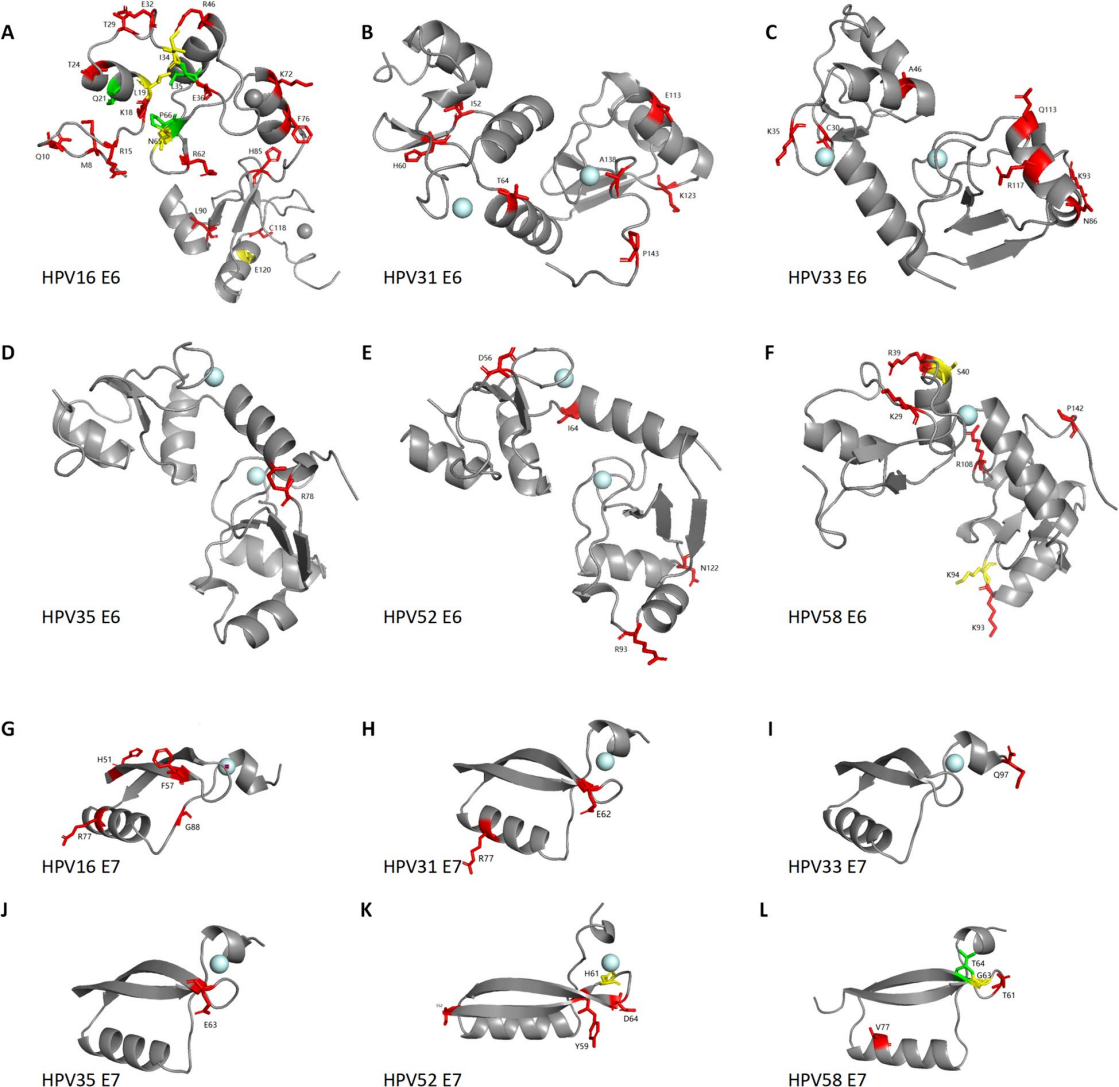
3D structural models of α-9 HPV E6 or E7 proteins

Nucleotide nonsynonymous substitution changes the amino acid composition, which affects the structure and function of proteins. Our analysis showed that α-9 E6 and E7 are composed of residues 148–158 and 97–99, respectively. The template-target pairwise sequence alignment for α-9 HPV E6 and E7. More details are shown in Table S1 and Figures S3-S4. All amino acid substitutions in E6 and E7 are shown in Fig. 3. As depicted in Figure S5, six E6 or E7 proteins of α-9 genus HPV are highly homologous, so their correlations are included in our research. As shown in Fig. 3, the majority of amino acid substitutions are located on the outer edge of E6 or E7 proteins and near the zinc granule, which is situated in the active site. Interestingly, we found that the 93rd residue is not only a common nonsynonymous mutation in the E6 region but also a positive selection site.

We selected the HPV variant with the highest infection rate in each type for homology modelling. The template coverage for the E6 protein of α-9 HPV included most of the protein sequence, no involving only a short N-terminal stretch (see Figure S5, A-F for the homology models and Figure S3 for the template-target pairwise sequence alignments). However, the template coverage for the E7 protein was low, excluding approximately 50 residues at the N-terminal stretch (see Figure S5, G-L for the homology models and Figure S4 for the template-target pairwise sequence alignments).

### Prediction of linear and conformational B-cell epitopes

The predicted linear B-cell epitopes of α-9 HPV E6 or E7 proteins are presented in Table 4. Conformational B-cell epitopes were predicted from protein tertiary structure models using ElliPro. Amino acid residues, the number of residues, the sequence location and the PI score of the predicted conformational epitopes are given in Table 5, and the graphical depiction of these epitopes are provided in Figure S6. Immunoinformatics predicted 57 potential linear and 59 conformational B-cell epitopes, many of which are also predicted as CTL epitopes (Tables 4, 5 and 6).

**Table 4:** Predicted linear B-cell epitopes of E6 and E7 proteins of α-9 genus HPV

Target Protein	No	Start	End	Peptide^a^	Length
HPV16 E6	1	19	25	LPQLCT**E**	7
	2	32	43	EIILECVYCKQQ	12
	3	55	61	RDLC**IVY**	7
	4	67	77	YAVCDKCL**KFY**	11
	5	84	90	RHYCYSL	7
	6	101	120	K**PLCDLLIRC**INCQKPLCPE	20
	7	144	149	MSCCRS	6
HPV16 E7	1	21	27	**DLY**CYEQ	7
	2	52	62	YNIVTFCCKCD	11
	3	64	73	TLRLCVQSTH	10
HPV31 E6	1	14	21	ELSSALEI	8
	2	27	35	RLNCVYCK**G**	9
	3	60	73	HGVCTKCL**RFYSKV**	14
	4	80	86	RYSVYGT	7
	5	97	113	CDLLIRCITCQRPLCPE	17
HPV31 E7	1	9	15	QDYVLDL	7
	2	21	27	**DLY**CYEQ	7
	3	55	62	VTFCCQCE	8
	4	64	72	TLRLCVQST	9
	5	88	94	GIVCPNC	7
HPV33 E6	1	13	19	HDLCQ**AL**	7
	2	27	36	ELQCVECK**KP**	10
	3	48	54	ADLTVVY	7
	4	62	70	ICKLCL**RFL**	9
	5	100	113	LIRCIICQRPLCPQ	14
HPV33 E7	1	9	16	KEYVLDLY	8
	2	21	27	DLYCYEQ	7
	3	52	62	**YY**IVTCCHTCN	11
	4	64	71	TVRLCVNS	8
HPV35 E6	1	24	35	HEICLNCVYCKQ	12
	2	43	54	YDFACYDLCIVY	12
	3	60	70	YGVCMKCL**KFY**	11
	4	94	114	KQLCHLLIRCITCQKPLCPVE	21
HPV35 E7	1	9	15	QDYVLDL	7
	2	21	30	**DLY**CYEQLCD	10
	3	56	63	VTSCCKCE	8
	4	65	73	TLRLCVQST	9
	5	89	95	**GIV**CPGC	7
HPV52 E6	1	13	21	HELCEVLEE	9
	2	25	35	EIRLQCVQCK**K**	11
	3	47	53	FTDLR**IV**	7
	4	60	71	YGVCIMCL**RFLS**	12
	5	78	83	HYQYSL	6
	6	100	112	TIRCIICQTPLCP	13
HPV52 E7	1	10	15	DYILDL	6
	2	22	27	**LH**CYEQ	6
	3	54	73	**YYIVTY**CHSCDSTLRLCIHS	20
HPV58 E6	1	13	21	HDLCQALET	9
	2	25	34	**EI**ELKCVECK	10
	3	43	53	YDFVFADLRIV	11
	4	60	72	FAVCKVCL**RLLSK**	13
	5	100	113	**LIRC**IICQRPLCPQ	14
	6	135	140	RCAVCW	6
HPV58 E7	1	10	16	**EY**ILDLH	7
	2	21	30	DLFCYEQLCD	10
	3	53	63	YYIVTCCYTCG	11
	4	65	71	TVRLCIN	7

^a^ residues highlighted in bold were also predicted to be CTL epitopes

**Table 5:** ElliPro predicted conformational B-cell epitopes of E6 and E7 proteins of α-9 genus HPV

Target protein	No	Residues^a^	Number of residues	PI score^b^
HPV16 E6	1	T6, A7, M8, F9, Q10, D11, P12, Q13, E14, R15, T24, E25, L26, Q27, T28, T29, I30, H31, E32, I33, R46, Y50	22	0.737
	2	D63, G64, N65	3	0.722
	3	Q123, L126, D127	3	0.715
	4	**T93, E96, Q97**, N100, K101, **P102, L103, C104, D105, L106, L107, R109, C110**, I111, N112, C113, Q114, K115, P116, L117, C118, P119, E120, R142, C143, M144, S145, C146, C147, R148, S149, S150	32	0.67
	5	R124, K128, K129, Q130	4	0.662
	6	F76, I80, S81, E82	4	0.564
	7	**Y39**, C70, D71, K72, C73, **K75**	6	0.56
	8	E36, C37, V38, C40, K41, Q42	6	0.555
HPV16 E7	1	**A50, H51**, Q70, S71, T72, H73, V74, R77	8	0.778
	2	G85, T86, L87	3	0.619
	3	V90, C91, P92, I93, C94, S95, Q96, K97, P98	9	0.599
HPV31 E6	1	R141, R142, P143, R144, T145, E146, T147, Q148, V149	9	0.852
	2	M1, F2, K3, N4, P5, A6, E7, R8, P9, R10, **S16, S17, A18, L19, E20, I21, P22, Y23**, D24, E25, **T38, E39**, D56, D57, T58	25	0.757
	3	V73, F76, R77, W78	4	0.743
	4	**R80, Y81, S82, V83, Y84**, G85, T86, T87, L88, E89, K90, L91, T92, N93, K94, G95, C97, D98, L99, K122, R124, G129	22	0.7
	5	Q116, R117, L119, D120, K121, K123, W140	7	0.638
	6	C111, P112, E113	3	0.517
HPV31 E7	1	S50, N51, Q70	3	0.91
	2	T72, Q73, V74, R77	4	0.708
	3	G85, S86, F87	3	0.59
	4	C58, C59, Q60, C61, E62, S63, **V90**, C91, P92, N93, C94, S95, T96, R97	14	0.542
HPV33 E6	1	G85, N86, T87, E89, Q90, T91, V92, K93, K94, P95, N97, E98, C111, P112, Q113	15	0.755
	2	M1, F2, Q3, D4, T5, E6, E7, K8, P9, T11, D14, C16, Q17, A18, L19, E20, T21, T22, I23, H24, N25, I26, E27, L28, E56, G57, N58	27	0.728
	3	**F69, I73, Y76, R77**, R144, R145, E146, T147	8	0.722
	4	Y84, K116, V119, D120, L121, N122, R124	7	0.71
	5	Y79, N80, Y81	3	0.651
	6	Q29, C30, V31, E32, C33, K34, **K35, P36, L37**	9	0.509
HPV33 E7	1	**A50, D51**, N70, S71, T72, A73, S74, R77, **Q81**	9	0.734
	2	G85, T86, V87	3	0.632
HPV35 E6	1	Q116, L119, E120, K122	4	0.776
	2	G85, E86, T87, E89, K90, Q91, C92, N93, K94, Q95, C97, H98, C111, P112, V113	15	0.751
	3	**F69, I73, Y76, R77, R78**, W140, K141, P142, T143, R144, R145, E146, T147	13	0.723
	4	M1, F2, Q3, D4, P5, A6, E7, R8, P9, D14, C16, N17, E18, V19, E20, E21, S22, I23, H24, E25, I26, C27, L28, N29, C30, C33, K34, Q35, E36, L37, E56, G57, Q58	33	0.702
	5	R80, Y81, G129	3	0.592
HPV35 E7	1	**S51, N52**, Q71, S72, T73, H74, I75, D76, R78, **K79**	10	0.733
	2	G86, T87, F88	3	0.632
	3	C59, C60, K61, C62, E63, A64, V91, C92, P93, G94, C95, S96, Q97, R98	14	0.531
HPV52 E6	1	G85, K86, T87, E89, E90, R91, V92, R93, K94, **P95, S97, E98**	12	0.804
	2	M1, F2, E3, D4, P5, A6, T7, R8, P9, T11, H13, E14, C16, E17, V18, L19, E20, E21, S22, V23, H24, E25, I26, D56, N57, N58	26	0.743
	3	**Y84**, E116, V119, N120, A121, N122, R124	7	0.707
	4	**F69, I73, Y76, R77**, C139, P142, R143, P144, V145, T146, Q147	11	0.672
	5	Y79, Q80, Y81	3	0.633
	6	R27, E36, L37	3	0.553
	7	C111, P112, E113	3	0.542
	8	Q29, C30, Q32, C33, K34, **K35**	6	0.535
HPV52 E7	1	S52, N53, H72	3	0.887
	2	T74, A75, T76, R79, **Q83**	5	0.694
	3	L86, G87, T88, L89	4	0.66
	4	C60, H61, S62, C63, D64, S65, **V92,** C93, P94, G95, C96, A97, R98	13	0.565
HPV58 E6	1	K116, V119, D120, L121, N122	5	0.788
	2	**I73, Y76, R77**, R144, R145, Q146, T147	7	0.741
	3	G85, D86, T87, E89, Q90, T91, L92, K93, K94, C95, N97, E98, I99, C111, P112, Q113	16	0.723
	4	M1, F2, Q3, D4, A5, E6, E7, K8, P9, T11, H13, D14, C16, Q17, A18, L19, E20, T21, S22, V23, H24, E25, I26, E27, L28, R55, D56, G57, N58	29	0.715
	5	Y79, N80, Y81	3	0.637
	6	K29, C30, V31, E32, C33, K34,** K35, T36, L37**	9	0.519
	7	C139, P142, R143	3	0.506
HPV58 E7	1	C92, P93, S94, A96, Q97, Q98	6	0.76
	2	A49, T50, A51, N52, N71, S72	6	0.727
	3	T73, T75, R78, Q82, M85, G86, T87, C88	8	0.698

^a^Residuea highlighted in bold were also predicted to be CTL epitopes

^b^Protrusion Index of ElliPro; a higher value indicates a higher probability for a discontinuous B-cell epitope

**Table 6:** NetCTL predicted CTL epitopes of E6 and E7 proteins of α-9 genus HPV

Target protein	MHC	Sequence position	Peptide sequence	Predicted MHC binding affinity	Rescale binding affinity	C terminal cleavage affinity	TAP transport efficiency	Predictions score
HPV16 E6	HLA-A*02	25	ELQTTIHEI	0.4989	1.1941	0.7786	0.328	1.3272
		18	KLPQLC**TEL**	0.347	0.8303	0.9987	1.198	1.04
		89	SLYG**T**TL**EQ**	0.2974	0.7118	0.9848	0.102	0.8646
		79	KISEYRHYC	0.3513	0.8408	0.0145	0.3	0.858
		29	TIHEIILEC	0.3209	0.7679	0.0407	0.162	0.7821
		102	PLCDLLIRC	0.325	0.7778	0.0166	-0.336	0.7635
	HLA-B*62	42	**QQ**LLRREVY	0.5208	1.3317	0.9495	3.132	1.6307
		59	IVYRDGNPY	0.4136	1.0577	0.9968	3.266	1.3705
		53	AFRDLCIVY	0.3355	0.8578	0.995	3.18	1.166
		75	KFYSKISEY	0.3131	0.8006	0.9984	3.434	1.1221
		44	LL**R**REV**Y**DF	0.3313	0.8471	0.9432	2.558	1.1164
		80	**ISE**YRHYCY	0.2849	0.7284	0.9933	2.945	1.0247
		83	YRHYCYSLY	0.2637	0.6743	0.8945	2.974	0.9572
		31	HEIILECVY	0.2503	0.6399	0.9569	2.776	0.9223
		1	MHQKR**TAMF**	0.2449	0.6262	0.9927	2.555	0.9028
		91	YG**T**TL**EQQ**Y	0.2207	0.5644	0.9946	2.65	0.846
HPV16 E7	HLA-A*02	11	YMLDLQPET	0.5367	1.2845	0.2388	-0.753	1.2826
		82	LLM**GTL**GIV	0.4531	1.0842	0.9111	0.264	1.2341
		7	TLHEYMLDL	0.4281	1.0245	0.9969	0.838	1.2159
		78	TLEDLLM**GT**	0.3651	0.8738	0.0166	-0.719	0.8403
	HLA-B*62	15	LQPETT**DLY**	0.5143	1.315	0.9981	2.85	1.6073
		43	GQAEPDR**AH**	0.5055	1.2925	0.0108	-0.468	1.2708
HPV31 E6	HLA-A*02	11	KLHELSSAL	0.4775	1.1428	0.9991	1.243	1.3549
	HLA-B*62	76	FRWYRYSVY	0.4181	1.0691	0.998	3.041	1.3709
		11	KLHELSSAL	0.358	0.9154	0.9991	1.243	1.1274
		15	LSSALEIPY	0.3315	0.8476	0.8163	2.798	1.1099
		46	AFTDLTIVY	0.3129	0.8001	0.9991	3.095	1.1047
		35	**G**QL**TE**TEVL	0.3172	0.8112	0.977	0.97	1.0062
		68	RFYSKVSEF	0.2382	0.6091	0.7779	3.181	0.8848
HPV31 E7	HLA-A*02	81	ELLM**GSF**GI	0.4221	1.0102	0.9178	0.319	1.1639
		7	TLQDYVLDL	0.407	0.9741	0.9982	0.776	1.1626
		82	LLM**GSF**GI**V**	0.3964	0.9487	0.9718	0.236	1.1062
		11	YVLDLQPEA	0.3693	0.8837	0.3393	-0.538	0.9077
		78	ILQELLM**GS**	0.4204	1.0062	0.0116	-2.103	0.9028
	HLA-B*62	15	LQPEATDLY	0.4592	1.1742	0.9983	2.85	1.4665
		79	LQELLM**GSF**	0.4901	1.2531	0.1649	2.479	1.4018
		3	GETPTLQDY	0.2993	0.7653	0.9989	2.706	1.0505
		44	QAEPDT**SN**Y	0.2749	0.703	0.9951	2.816	0.993
HPV33 E6	HLA-A*02	18	ALETTIHNI	0.5326	1.2746	0.9869	0.505	1.4479
		11	TLHDLCQAL	0.4743	1.135	0.9948	1.115	1.34
	HLA-B*62	37	**L**QRSEVYDF	0.4917	1.2572	0.8544	2.518	1.5112
		46	AFADLTVVY	0.4194	1.0723	0.9991	3.148	1.3796
		76	YRHYNYSVY	0.3882	0.9927	0.9987	2.974	1.2912
		52	VVYREGNPF	0.3669	0.9381	0.3607	2.932	1.1388
		73	**I**SE**YR**HYNY	0.3131	0.8007	0.875	2.945	1.0792
		11	TLHDLCQAL	0.3151	0.8058	0.9948	1.115	1.0107
		35	**KPL**QRSEVY	0.2724	0.6966	0.9966	2.846	0.9884
		68	RFLSKISEY	0.1989	0.5086	0.9951	3.402	0.8279
HPV33 E7	HLA-A*02	82	LLMGTVNIV	0.54	1.2924	0.9954	0.354	1.4594
		7	TLKEYVLDL	0.3347	0.8009	0.9988	0.806	0.991
		81	**Q**LLM**GTV**NI	0.3349	0.8015	0.5909	0.536	0.917
	HLA-B*62	44	QAQPAT**ADY**	0.4455	1.1392	0.9926	2.928	1.4345
		45	AQPAT**ADYY**	0.3146	0.8043	0.2182	3.08	0.9911
		3	GHKPTL**KEY**	0.224	0.5728	0.9989	2.883	0.8668
		79	IQ**Q**LLM**GTV**	0.2777	0.7101	0.57	0.391	0.8151
		15	**LY**PEPTDLY	0.1844	0.4715	0.9989	3.039	0.7732
HPV35 E6	HLA-A*02	11	KLHDLCNEV	0.6042	1.4459	0.9989	0.419	1.6167
		18	EVEESIHEI	0.4061	0.9719	0.993	0.466	1.1441
	HLA-B*62	2	FQDPAERPY	0.5554	1.4203	0.9729	2.765	1.7045
		52	IVYREGQPY	0.4651	1.1892	0.948	3.266	1.4947
		37	**L**QRSEV**YDF**	0.4917	1.2572	0.6145	2.496	1.4741
		76	YRRYRYSVY	0.3898	0.9967	0.994	2.993	1.2955
		35	**Q**ELQRSEV**Y**	0.3691	0.9438	0.9956	2.921	1.2392
		46	ACYDLCIVY	0.3243	0.8291	0.9988	3.171	1.1375
		68	KFYSKISEY	0.3131	0.8006	0.9982	3.434	1.122
		24	HEICLNCVY	0.2112	0.54	0.9733	2.776	0.8248
		40	SEVYDFACY	0.1894	0.4842	0.8797	3.052	0.7688
		73	ISEYRRYRY	0.1899	0.4855	0.9067	2.945	0.7687
HPV35 E7	HLA-A*02	7	TLQDYVLDL	0.407	0.9741	0.9983	0.931	1.1703
		83	LLMGTFGIV	0.4069	0.9738	0.9965	0.264	1.1365
		82	DLLMGTFGI	0.3616	0.8654	0.9784	0.089	1.0166
		79	**K**LEDLLM**GT**	0.4062	0.972	0.032	-0.591	0.9473
		11	YVLDLEPEA	0.3599	0.8612	0.3088	-0.538	0.8807
	HLA-B*62	45	QAKPDT**SN**Y	0.2792	0.7139	0.996	2.958	1.0112
		3	GEITTLQDY	0.2491	0.6369	0.9984	2.656	0.9195
		15	LEPEAT**DLY**	0.1899	0.4856	0.9972	2.639	0.7672
HPV52 E6	HLA-A*02	18	VLEESVHEI	0.5803	1.3887	0.9965	0.305	1.5535
		45	FLFTDLR**IV**	0.4719	1.1294	0.8378	0.452	1.2777
		11	TLHELCEVL	0.3916	0.9372	0.9982	1.115	1.1427
		95	**P**L**SE**ITIRC	0.3666	0.8772	0.3184	-0.349	0.9075
	HLA-B*62	52	IVYRDNNPY	0.3531	0.903	0.9256	3.405	1.2121
		37	**L**QRREVYKF	0.3499	0.8948	0.8351	2.496	1.1448
		46	LFTDLR**IVY**	0.3279	0.8385	0.9983	2.955	1.136
		73	ISEYRHYQY	0.3169	0.8104	0.9968	2.945	1.1071
		76	YRHYQYSLY	0.3	0.7671	0.998	2.974	1.0655
		35	**KEL**QRREVY	0.2843	0.727	0.8219	3.015	1.0011
		68	RFLSKISEY	0.1989	0.5086	0.9984	3.402	0.8284
		11	TLHELCEVL	0.241	0.6163	0.9982	1.115	0.8217
HPV52 E7	HLA-A*02	84	MLLGTLQVV	0.5764	1.3794	0.9963	0.35	1.5464
		83	QML**LGTL**QV	0.3515	0.8411	0.9985	0.418	1.0118
		11	YILDLQPET	0.3721	0.8905	0.2146	-0.718	0.8868
		7	TIKDYILDL	0.2586	0.619	0.9985	1.214	0.8294
	HLA-B*62	51	TSNYYIVTY	0.2515	0.643	0.9991	3.004	0.9431
		15	LQPETTD**LH**	0.3372	0.8623	0.4693	-0.61	0.9022
		47	AEQATSN**YY**	0.2249	0.5752	0.9118	2.852	0.8545
		81	LQQMLLGTL	0.236	0.6035	0.9513	1.012	0.7968
		46	QAEQATSNY	0.2083	0.5326	0.6621	2.816	0.7727
HPV58 E6	HLA-A*02	18	ALETSVHEI	0.4968	1.1888	0.9966	0.505	1.3636
		11	TLHDLCQAL	0.4743	1.135	0.9986	1.115	1.3406
		45	FVFADLRIV	0.3196	0.7648	0.7944	0.331	0.9005
		82	SLYGDTLEQ	0.2825	0.676	0.78	0.102	0.7981
		95	CLNEILIRC	0.3211	0.7684	0.0464	0	0.7754
	HLA-B*62	37	LQRSEV**YDF**	0.4917	1.2572	0.2985	2.673	1.4356
		35	**KTL**QRSEV**Y**	0.3963	1.0134	0.9144	3.129	1.307
		68	RLLSKISEY	0.3612	0.9235	0.9981	3.254	1.2359
		46	VFADLRIVY	0.358	0.9155	0.985	3.066	1.2165
		52	IVYRDGNPF	0.3659	0.9357	0.6274	3.015	1.1806
		73	ISEYRHYNY	0.3131	0.8007	0.9961	2.945	1.0973
		76	YRHYNYSLY	0.2908	0.7437	0.9837	2.974	1.04
		11	TLHDLCQAL	0.3151	0.8058	0.9986	1.115	1.0113
HPV58 E7	HLA-A*02	83	LL**MGTC**TIV	0.5007	1.1981	0.9983	0.354	1.3656
		7	TLREYILDL	0.3818	0.9138	0.9989	0.857	1.1065
		82	QLLMGTCTI	0.3692	0.8834	0.992	0.536	1.059
		60	YTCGTTVRL	0.2937	0.7029	0.9988	0.915	0.8985
		79	TLQ**Q**LL**MGT**	0.3299	0.7896	0.0188	-0.607	0.7621
	HLA-B*62	45	QAQP**ATAN**Y	0.4122	1.054	0.9726	2.928	1.3463
		46	AQP**ATAN**YY	0.373	0.9539	0.371	3.08	1.1635
		3	GNNPTLR**EY**	0.3069	0.7846	0.9956	2.631	1.0655
		83	LL**MGTC**TIV	0.2591	0.6626	0.9983	0.354	0.83
		52	NYYIVTCCY	0.1915	0.4896	0.9954	3.567	0.8173

^a^Amino acids highlighted in bold were also predicted as B-cell antigenic sites (linear and/or conformational)

^b^Prediction score threshold > 0.75

### Prediction of cytotoxic T-cell epitopes

Based on the literature, we selected the HLA-A*02 and HLA-B*62 supertypes for NetCTL epitope prediction, as well as CTL epitopes that overlap with the sites for the predicted B-cell epitopes (linear and/or conformational). The predicted CTL epitope 9-mer peptides with their predicted MHC-I binding affinity, rescaled binding affinity, proteasomal C-terminal cleavage affinity, and TAP transport efficiency are indicated in Table 6, with an overall prediction score threshold of 0.75.

## Discussion

Cervical cancer remains a major challenge for women’s health, with approximately 600,000 new cases and 340,000 deaths worldwide every year [1, 26]. High-risk HPV genotypes are the main cause of cervical cancer, which can be largely prevented through HPV vaccination and cervical screening. However, the cervical cancer burden for women remains heavy in China, with only 3% of women aged 9–45 years receiving complete HPV vaccination [27, 28]. Although prophylactic vaccines are effective at protecting against approximately 90% of HPV infections, their benefits in eliminating preexisting infection are limited. Therefore, potential therapeutic vaccines need to be developed for treatment of persistent HPV infection or cervical lesions, with the goal of activating adaptive immune responses by presenting viral antigen peptides to the immune system. However, because the genetic variations of HPV genotypes show some degree of geographical differences, it is recommended that the ideal therapeutic vaccine be based on a local type.

The Taizhou area is located along the central coast of Zhejiang Province in China, with high prevalence and pathogenicity of α-9 HPV (HPV 16, 31, 33, 35, 52, 58), especially HPV52 and 58 [20]. According to our previous findings, the odds ratio (OR) for CIN2 + in women infected with α-9 HPV is 3.2 when compared to women infected with α-5, α-6, or α-7 [7]. The genetic variation of *E6* or *E7* genes may correlate highly with cancer risk in Taizhou [21–23]. Therefore, E6 or E7 molecules might be regarded as ideal targets for HPV therapeutic vaccine development and cervical cancer treatment. The purpose of this study was to provide data for effective prevention and treatment of HPV-induced cervical lesions in Taizhou by analysing the phylogenetic tree and epitope prediction of α-9 HPV E6 or E7.

In Taizhou, we found that the most common nucleotide mutations observed in the *E6* gene were T178G (D32E, 192/298, positive selection) of HPV16; C520T (I138V, 63/149) of HPV31; A387C (K93N, 45/185, positive selection) of HPV33; T341C (W78R, 122/123) of HPV35; A379G (K93R, 313/325, positive selection) of HPV52; and A388C (K93N, 87/199, positive selection) of HPV58. Most high-frequency nonsynonymous mutations are positive selection sites, which contribute to HPV adaptation. In addition, their corresponding genetic variants have been widespread in Taizhou, including MT681266 (38.9%, 116/298, HPV16), OR540577 (24.8%, 37/149, HPV31), OQ672675 (20%, 37/185, HPV33), OR540579 (62.6%, 77/123, HPV35), ON529581 (60.9%, 198/325, HPV52), MH348927 (22.1%, 44/199, HPV58). Interestingly, we found that the 93rd residue is not only a common nonsynonymous mutation in the E6 region but also a positive selection site, which may have evolutionary significance in rendering HPV33, 52, and 58 adaptive to their environment. Notably, three amino acid substitutions at the 93rd residue were detected in HPV52 E6: K93R (96.3%), K93G (2.5%), and K93Q (2.2%). In addition, the K93N substitution was observed in both HPV33 and HPV58. These 93rd-amino acid substitutions may lead to differences in the ability of these viruses to bind to p53, thus affecting their carcinogenicity [29]. The D32E substitution in HPV16 E6 has been confirmed to be associated with cancer risk, possibly because the amino acid substitution affects the E6-p53 interaction [30].

In the *E7* gene, the most common nucleotide mutation observed was A647G (N29S, 195/298, positive selection) of HPV16. Similarly, there were three amino acid substitutions at the 29th residue in HPV16 E7: N29S (65.44%), N29H (9.40%), and N29T (0.34%). In addition, all HPV58 E7 with G63S carries T20I, and all HPV58 E7 samples with G63D carry G41R. In our previous study, it was reported that HPV58 E7 T20I/G63S substitutions increase risk of HPV carcinogenesis [24]. Boon et al. [31] reported that T20I/G63S substitutions possess greater ability to degrade Rb, immortalize, and transform primary cells.

Consistently with our selective pressure analysis, positive selection sites for 32E in HPV16 E6, 29S in HPV16 E7 were found in Kunming, Southwest of China [32]. Positive selection sites for 93K in HPV33 E6, 45A, 97Q in HPV33 E7 were found in Sichuan province, Southwest of China [33]. Notably, positive selection site for 63G in HPV58 E7, which has been widely reported in other regions of China, including Sichuan province [33] and Yunnan province [34, 35] in Southwest China, Hubei province in central China [36], as well as the present study (Southeast China). However, there were no reports of positive selection sites for HPV58 E6 in China [33–36]. No positive selection sites for HPV16 E6 and E7 genes have been reported in central China [37]. No positive selection sites for HPV52 E6 and E7 genes have been reported in central and Southwest China [38, 39]. In addition, selective pressure analysis from non-Asian population showed different results, with 10R, 14H, 83V in HPV E6 and 85G in HPV16 E7 under positive selective pressure [40, 41]. Therefore, these results indicate that genetic variations among HPV types may lead to biological advantages through fixed mutations in their genomes and that even small variations might lead to minor adaptive improvements. Furthermore, the genetic variations of HPV may differ in terms of infectivity, carcinogenic potential, and host immune response. Therefore, the data provided in this study may have significant implications for understanding the biological differences of HPVs in Taizhou, as well as for developing local therapeutic vaccines.

HLA participates in the local immune response of viral infection through its target recognition function, blocking HPV infection or preventing tumour cell invasion and metastasis. However, a minority of infected cells can escape host immune surveillance, causing persistent HPV infection. Immunoinformatics provides new strategies for identifying ideal epitopes for HPV therapeutic vaccine targets. Our predicted T- and B-cell epitopes may be used for development of vaccines targeting specific HPV variants, and our results suggest that amino acid substitution may influence these epitopes. For example, the prediction score of the HPV16 E6 CTL epitope 25-33ELQTTIHDI was 1.0904, and because of the D32E substitution the score of the epitope 25-33ELQTTIHEI increased to 1.3272; the HPV16 E6 predicted epitope 31-39HEIILECVY became a new epitope because of the mutation D32E. Therefore, the substitution of the positive selection site D32E in HPV16 E6 influences its antigenic epitopes, which may make it more difficult to detect by the immune system, thereby enhancing adaptability of HPV to the environment. In addition, Li et al. [37] suggested that non-conservative substitutions of amino acids should be fully considered when developing therapeutic vaccines, such as H31Y, D32N, D32E, I34M, L35V, E36Q, L45P, N65S, and K75T in HPV16 E6. Chen et al. [33] suggested that K93N in HPV33 E6, Q97L in HPV33 E7, R145K in HPV58 E6, and T20I in HPV58 E7 belong to ideal B-cell epitopes.

In addition, our results suggest that the CTL epitope of HPV16 E6 is 75-83KFYSKISEY; homologous epitopes were identified in HPV31 E6 68-76RFYSKVSEF, HPV33 E6 68-76RFLSKISEY, HPV35 E6 68-76KFYSKISEY, HPV52 E6 68-76RFLSKISEY, and HPV58 E6 68-76RLLSKISEY. Homologous epitopes were also identified in HPV16 E7 11-19YMLDLQPET, HPV31 E7 11-19YVLDLQPEA, HPV35 E7 11-19YVLDLEPEA, and HPV52 E7 11-19YILDLQPET. These E7 epitopes are restricted to HLA-A*02, which is the first noteworthy allele of the HPV-restricted epitope [42–44]. Riemer et al. [45] found that CTLs can recognize and lyse target cells presenting HPV16 E7 11-19YMLDLQPET. However, further experiments are required to validate the potential HPV therapeutic vaccines predicted through immunoinformatics.

## Conclusions

In summary, this is the first almost comprehensive study to explore the genetic variations, phylogenetics, positive selection sites, and antigenic epitopes of α-9 HPV E6 and E7 molecules in Taizhou, China, and the results will be helpful for local HPV therapeutic vaccine development.

### Supplementary Information

Supplementary Material 1.

Supplementary Material 2.

Supplementary Material 3.

Supplementary Material 4.

Supplementary Material 5.

Supplementary Material 6.

Supplementary Material 7.

Supplementary Material 8.

Supplementary Material 9.

## Data Availability

All data generated during this study are included in this published article. All sequences of α-9 HPV E6 and E7 genes are available in the GenBank database with the accession codes of MT681266 to MT681329, OR540563 to OR540578, OQ672665 to OQ672679, OR540579 to OR540583, ON529577 to ON529603, and MH348918 to MH348942. The links are https://www.ncbi.nlm.nih.gov/nuccore/MT681266 ~ https://www.ncbi.nlm.nih.gov/nuccore/MH348942.

## References

[1] Cohen PA, Jhingran A, Oaknin A, Denny L (2019). Cervical cancer. Lancet.

[2] Alejo M, Alemany L, Clavero O, Quiros B, Vighi S, Seoud M (2018). Contribution of Human papillomavirus in neuroendocrine tumors from a series of 10,575 invasive cervical cancer cases. Papillomavirus Res.

[3] Matsumoto K, Oki A, Furuta R, Maeda H, Yasugi T, Takatsuka N (2011). Predicting the progression of cervical precursor lesions by human papillomavirus genotyping: a prospective cohort study. Int J Cancer.

[4] Ferenczy A, Franco E (2002). Persistent human papillomavirus infection and cervical neoplasia. Lancet Oncol.

[5] Arroyo Mühr LS, Lagheden C, Hassan SS, Eklund C, Dillner J (2023). The International Human Papillomavirus Reference Center: Standardization, collaboration, and quality assurance in HPV research and diagnostics. J Med Virol.

[6] Chen Z, Schiffman M, Herrero R, Desalle R, Anastos K, Segondy M (2011). Evolution and taxonomic classification of human papillomavirus 16 (HPV16)-related variant genomes: HPV31, HPV33, HPV35, HPV52, HPV58 and HPV67. PLoS One.

[7] Xu H, Lin A, Shao X, Shi W, Zhang Y, Yan W (2016). Diagnostic accuracy of high-risk HPV genotyping in women with high-grade cervical lesions: evidence for improving the cervical cancer screening strategy in China. Oncotarget.

[8] Tommasino M (2014). The human papillomavirus family and its role in carcinogenesis. Semin Cancer Biol.

[9] Fehrmann F, Laimins LA (2003). Human papillomaviruses: targeting differentiating epithelial cells for malignant transformation. Oncogene.

[10] Tsakogiannis D, Gortsilas P, Kyriakopoulou Z, Ruether IG, Dimitriou TG, Orfanoudakis G (2015). Sites of disruption within E1 and E2 genes of HPV16 and association with cervical dysplasia. J Med Virol.

[11] Hu Z, Zhu D, Wang W, Li W, Jia W, Zeng X (2015). Genome-wide profiling of HPV integration in cervical cancer identifies clustered genomic hot spots and a potential microhomology-mediated integration mechanism. Nat Genet.

[12] Cancer Genome Atlas Research Network; Albert Einstein College of Medicine; Analytical Biological Services; Barretos Cancer Hospital; Baylor College of Medicine; Beckman Research Institute of City of Hope; Buck Institute for Research on Aging (2017). Integrated genomic and molecular characterization of cervical cancer. Nature.

[13] Yang A, Farmer E, Wu TC, Hung CF (2016). Perspectives for therapeutic HPV vaccine development. J Biomed Sci.

[14] Gomes D, Silvestre S, Duarte AP, Venuti A, Soares CP, Passarinha L (2021). In silico approaches: a way to unveil novel therapeutic drugs for cervical cancer management. Pharmaceuticals (Basel).

[15] Mukherjee AG, Wanjari UR, Gopalakrishnan AV, Kannampuzha S, Murali R, Namachivayam A (2022). Exploring the Molecular Pathogenesis, Pathogen Association, and Therapeutic Strategies against HPV Infection. Pathogens.

[16] Jhunjhunwala S, Hammer C, Delamarre L (2021). Antigen presentation in cancer: insights into tumour immunogenicity and immune evasion. Nat Rev Cancer.

[17] Markowitz LE, Unger ER (2023). Human papillomavirus vaccination. N Engl J Med.

[18] Mo Y, Ma J, Zhang H, Shen J, Chen J, Hong J (2022). Prophylactic and therapeutic HPV vaccines: current scenario and perspectives. Front Cell Infect Microbiol.

[19] Tian Y, Hu D, Li Y, Yang L (2022). Development of therapeutic vaccines for the treatment of diseases. Mol Biomed.

[20] Xu HH, Lin A, Chen YH, Dong SS, Shi WW, Yu JZ (2017). Prevalence characteristics of cervical human papillomavirus (HPV) genotypes in the Taizhou area, China: a cross-sectional study of 37 967 women from the general population. BMJ Open.

[21] Dai MZ, Qiu Y, Di XH, Shi WW, Xu HH (2021). Association of cervical carcinogenesis risk with HPV16 E6 and E7 variants in the Taizhou area. China BMC Cancer.

[22] Yan ZY, Di XH, Qiu Y, Ying YY, Gan J, Xu HH (2023). Cervical carcinogenesis risk association of HPV33 E6 and E7 genetic variations in Taizhou, Southeast China. Virol J.

[23] Yang Z, He ZH, Zhang Y, Di XH, Zheng DF, Xu HH (2022). Genetic variability in the E6 and E7 oncogenes of HPV52 and its prevalence in the Taizhou area. China Virol J.

[24] Yu JH, Shi WW, Zhou MY, Liu JM, Han QY, Xu HH (2019). Genetic variability and oncogenic risk association of human papillomavirus type 58 E6 and E7 genes in Taizhou area. China Gene.

[25] Álvarez-Carretero S, Kapli P, Yang Z (2023). Beginner's guide on the use of PAML to detect positive selection. Mol Biol Evol.

[26] Sung H, Ferlay J, Siegel RL, Laversanne M, Soerjomataram I, Jemal A (2021). Global Cancer Statistics 2020: GLOBOCAN Estimates of Incidence and Mortality Worldwide for 36 Cancers in 185 Countries. CA Cancer J Clin.

[27] Wang H, Jiang Y, Wang Q, Lai Y, Holloway A (2023). The status and challenges of HPV vaccine programme in China: an exploration of the related policy obstacles. BMJ Glob Health.

[28] Rahangdale L, Mungo C, O'Connor S, Chibwesha CJ, Brewer NT (2022). Human papillomavirus vaccination and cervical cancer risk. BMJ.

[29] Ainsworth J, Thomas M, Banks L, Coutlee F, Matlashewski G (2008). Comparison of p53 and the PDZ domain containing protein MAGI-3 regulation by the E6 protein from high-risk human papillomaviruses. Virol J.

[30] Ai W, Wu C, Jia L, Xiao X, Xu X, Ren M (2022). Deep sequencing of HPV16 E6 Region reveals unique mutation pattern of HPV16 and predicts cervical cancer. Microbiol Spectr.

[31] Boon SS, Xia C, Lim JY, Chen Z, Law PTY, Yeung ACM (2020). Human papillomavirus 58 E7 T20I/G63S variant isolated from an East Asian population possesses high Oncogenicity. J Virol.

[32] Yang L, Yang H, Wu K, Shi X, Ma S, Sun Q (2014). Prevalence of HPV and variation of HPV 16/HPV 18 E6/E7 genes in cervical cancer in women in South West China. J Med Virol.

[33] Chen Z, Jing Y, Wen Q, Ding X, Wang T, Mu X (2017). E6 and E7 Gene Polymorphisms in Human Papillomavirus Types-58 and 33 Identified in Southwest China. PLoS One.

[34] Xi J, Chen J, Xu M, Yang H, Wen S, Pan Y (2018). The polymorphisms of LCR, E6, and E7 of HPV-58 isolates in Yunnan, Southwest China. Virol J.

[35] Yang L, Yang H, Chen J, Huang X, Pan Y, Li D (2014). Genetic variability of HPV-58 E6 and E7 genes in Southwest China. Infect Genet Evol.

[36] Yang Z, Zhang C, Luo P, Ye M, Gong Q, Mei B (2022). Genetic variability of E6 and E7 genes of human papillomavirus type 58 in Jingzhou, Hubei Province of central China. Virol J.

[37] Li T, Yang Z, Zhang C, Wang S, Mei B (2023). Genetic variation of E6 and E7 genes of human papillomavirus type 16 from central China. Virol J.

[38] Li S, Ye M, Chen Y, Gong Q, Mei B (2021). Genetic variation of E6 and E7 genes of human papillomavirus 52 from Central China. J Med Virol.

[39] Song Z, Cui Y, Li Q, Deng J, Ding X, He J (2021). The genetic variability, phylogeny and functional significance of E6, E7 and LCR in human papillomavirus type 52 isolates in Sichuan, China. Virol J.

[40] Carvajal-Rodríguez A (2008). Detecting recombination and diversifying selection in human alpha-papillomavirus. Infect Genet Evol.

[41] Bletsa G, Zagouri F, Amoutzias GD, Nikolaidis M, Zografos E, Markoulatos P (2021). Genetic variability of the HPV16 early genes and LCR. Present and future perspectives. Expert Rev Mol Med..

[42] He Y, Li J, Mao W, Zhang D, Liu M, Shan X (2018). HLA common and well-documented alleles in China. HLA.

[43] Yao Y, Huang W, Yang X, Sun W, Liu X, Cun W (2013). HPV-16 E6 and E7 protein T cell epitopes prediction analysis based on distributions of HLA-A loci across populations: an in silico approach. Vaccine.

[44] Chenzhang Y, Wen Q, Ding X, Cao M, Chen Z, Mu X (2017). Identification of the impact on T- and B-cell epitopes of human papillomavirus type-16 E6 and E7 variant in Southwest China. Immunol Lett.

[45] Riemer AB, Keskin DB, Zhang G, Handley M, Anderson KS, Brusic V (2010). A conserved E7-derived cytotoxic T lymphocyte epitope expressed on human papillomavirus 16-transformed HLA-A2+ epithelial cancers. J Biol Chem.

