# Supplementary material for: Phylogenetic analysis and antigenic epitope prediction for E6 and E7 of Alpha-papillomavirus 9 in Taizhou, China
Source: BMC Genomics. 2024 May 22;25:507. doi: 10.1186/s12864-024-10411-1 (PMC11110188; doi:10.1186/s12864-024-10411-1)
Supplement: Supplementary file 1 — Supplementary Material 1. [file 12864_2024_10411_MOESM1_ESM.docx]

**Table S1. Primers and PCR condition used for the molecular characterization of α-9 HPV *E6* and *E7* genes**

| **GenBank**^a^ | **Primer name** | **Sequence 5′ to 3′** | **Tm** | **Product size** | **Nucleotide sites**^b^ | **Number of amino acid residue**^c^ |
| --- | --- | --- | --- | --- | --- | --- |
| K02718 | HPV16 E6E7F | 5′-ACTAAGGGCGTAACCGAAAT-3′ | 57°C | 1061bp | nt23–1083, including *E6* gene nt83–559 and *E7* gene nt562–858 | E6:158, E7:98 |
|  | HPV16 E6E7R | 5′-TGCAGTAAACAACGCAT-3′ |  |  |  |  |
| J04353 | HPV31 E6E7F | 5′-AGGGAGTGACCGAAAGTGGT-3′ | 62°C | 1066bp | nt30–1095, including *E6* gene nt108–557 and *E7* gene nt560–856 | E6:149, E7:98 |
|  | HPV31 E6E7R | 5′-ATGTTCCTCCGCTTCCTGTG-3′ |  |  |  |  |
| M12732 | HPV33 E6E7F | 5′-AGGGTGTAACCGAAAGCGG-3’ | 60°C | 1164bp | nt31–1194, including *E6* gene nt109–558 and *E7* gene nt573–866 | E6:149, E7:97 |
|  | HPV33 E6E7R | 5′-TTGCAGCACGATCAACAACG-3’ |  |  |  |  |
| HQ537708 | HPV35 E6E7F | 5′-AGTGACCGAAAACGGTCGTA-3’ | 60°C | 944bp | nt19–962, including *E6* gene nt110–559 and *E7* gene nt562–861 | E6:149, E7:99 |
|  | HPV35 E6E7R | 5′-GGATCCCCCGTACGTCTACT-3’ |  |  |  |  |
| HQ537732 | HPV52 E6E7F | 5′-ACCCACAACCACTTTTTTTTAT-3′ | 60°C | 957bp | nt7916–930, including *E6* gene nt102–548 and *E7* gene nt553–852 | E6:148, E7:99 |
|  | HPV52 E6E7R | 5′-TTGCCTCTACTTCAAACCAGCC-3′ |  |  |  |  |
| NC001443 | HPV58 E6E7F | 5′-CGAAAACGGTCTGACCGAAA-3′ | 56°C | 968bp | nt42–1009, including *E6* gene nt110–559 and *E7* gene nt574–870 | E6:149, E7:98 |
|  | HPV58 E6E7R | 5′-TATCGTCTGCTGTTTCGTCC-3′ |  |  |  |  |
| ^a^ the accession number of HPV reference sequence in GenBank  ^b^ nt: nucleotide sites  ^c^ excluding the termination codons | | | | | |  |
