# Supplementary material for: Phylogenetic analysis and antigenic epitope prediction for E6 and E7 of Alpha-papillomavirus 9 in Taizhou, China
Source: BMC Genomics. 2024 May 22;25:507. doi: 10.1186/s12864-024-10411-1 (PMC11110188; doi:10.1186/s12864-024-10411-1)
Supplement: Supplementary file 2 — Supplementary Material 2. [file 12864_2024_10411_MOESM2_ESM.docx]

| **HPV genotypes** | | Overall population  (n=60259) | | | | | |
| --- | --- | --- | --- | --- | --- | --- | --- |
|  |  | Single infections (n=7698) | | Multipleinfections (n=2725) | | Overall infections (n=10423) | |
| **α-9** | **HPV52** | 1844 | 17.69% | 1088 | 10.44% | 2932 | 28.13% |
|  | **HPV58** | 1087 | 10.43% | 740 | 7.10% | 1827 | 17.53% |
|  | **HPV16** | 1021 | 9.80% | 638 | 6.12% | 1659 | 15.92% |
|  | **HPV33** | 312 | 2.99% | 322 | 3.09% | 634 | 6.08% |
|  | **HPV31** | 243 | 2.33% | 190 | 1.82% | 433 | 4.15% |
|  | **HPV35** | 166 | 1.59% | 164 | 1.57% | 330 | 3.17% |
| **α-7** | **HPV39** | 658 | 6.31% | 464 | 4.45% | 1122 | 10.76% |
|  | **HPV18** | 525 | 5.04% | 419 | 4.02% | 944 | 9.06% |
|  | **HPV59** | 345 | 3.31% | 338 | 3.24% | 683 | 6.55% |
|  | **HPV68** | 270 | 2.59% | 279 | 2.68% | 549 | 5.27% |
|  | **HPV45** | 107 | 1.03% | 116 | 1.11% | 223 | 2.14% |
| **α-6** | **HPV56** | 381 | 3.66% | 445 | 4.27% | 826 | 7.92% |
|  | **HPV66** | 328 | 3.15% | 312 | 2.99% | 640 | 6.14% |
| **α-5** | **HPV51** | 411 | 3.94% | 371 | 3.56% | 782 | 7.50% |

**Table S2. Prevalence of high-risk HPV genotypes in women (Taizhou, Zhejiang)**
