# Supplementary material for: Phylogenetic analysis and antigenic epitope prediction for E6 and E7 of Alpha-papillomavirus 9 in Taizhou, China
Source: BMC Genomics. 2024 May 22;25:507. doi: 10.1186/s12864-024-10411-1 (PMC11110188; doi:10.1186/s12864-024-10411-1)
Supplement: Supplementary file 3 — Supplementary Material 3. [file 12864_2024_10411_MOESM3_ESM.docx]

| Table S3. Analysis on the oncogenic risk association of α-9 HPV types | | | | | | |
| --- | --- | --- | --- | --- | --- | --- |
| **α-9 HPV (sub)lineages** | **Normal** | **CIN1** | **CIN2** | **CIN3** | **Cancer** | **Total** |
| **HPV16**^[21]^ |  |  |  |  |  |  |
| **A1** | **4****(6.2%)** | **2(9.1%)** | **1(3.2%)** | **2(3.3%)** | **1(6.7%)** | **10(5.2%)** |
| 16CNTZ32 | 3(4.6%) | 0 | 1(3.2%) | 1(1.6%) | 1(6.7%) | 6(3.1%) |
| 16CNTZ31 | 1(1.5%) | 1(4.5%) | 0 | 1(1.6%) | 0 | 3(1.5%) |
| 16CNTZ27 | 0 | 1(4.5%) | 0 | 0 | 0 | 1(0.5%) |
| **A2** | **8(12.3%)** | **3(13.6%)** | **5(16.1%)** | **5(8.2%)** | **2(13.3%)** | **23(11.9%)** |
| 16CNTZ35 | 3(4.6%) | 1(4.5%) | 0 | 2(3.3%) | 2(13.3%) | 8(4.1%) |
| 16CNTZ49 | 2(3.1%) | 0 | 1(3.2%) | 0 | 0 | 3(1.5%) |
| 16CNTZ36 | 0 | 0 | 1(3.2%) | 1(1.6%) | 0 | 2(1.0%) |
| 16CNTZ33 | 0 | 0 | 1(3.2%) | 0 | 0 | 1(0.5%) |
| 16CNTZ34 | 0 | 0 | 0 | 1(1.6%) | 0 | 1(0.5%) |
| 16CNTZ38 | 1(1.5%) | 0 | 0 | 0 | 0 | 1(0.5%) |
| 16CNTZ39 | 1(1.5%) | 0 | 0 | 0 | 0 | 1(0.5%) |
| 16CNTZ50 | 1(1.5%) | 0 | 0 | 0 | 0 | 1(0.5%) |
| 16CNTZ51 | 0 | 0 | 1(3.2%) | 0 | 0 | 1(0.5%) |
| 16CNTZ52 | 0 | 0 | 0 | 1(1.6%) | 0 | 1(0.5%) |
| 16CNTZ53 | 0 | 1(4.5%) | 0 | 0 | 0 | 1(0.5%) |
| 16CNTZ54 | 0 | 1(4.5%) | 0 | 0 | 0 | 1(0.5%) |
| 16CNTZ55 | 0 | 0 | 1(3.2%) | 0 | 0 | 1(0.5%) |
| **A3** | **5(7.7%)** | **5(22.7%)** | **3(9.7%)** | **6(9.8%)** | **0** | **19(9.8%)** |
| 16CNTZ40 | 5(7.7%) | 2(9.1%) | 2(6.5%) | 2(3.3%) | 0 | 11(5.7%) |
| 16CNTZ42 | 0 | 1(4.5%) | 1(3.2%) | 0 | 0 | 2(1.0%) |
| 16CNTZ41 | 0 | 0 | 0 | 1(1.6%) | 0 | 1(0.5%) |
| 16CNTZ43 | 0 | 1(4.5%) | 0 | 0 | 0 | 1(0.5%) |
| 16CNTZ44 | 0 | 1(4.5%) | 0 | 0 | 0 | 1(0.5%) |
| 16CNTZ45 | 0 | 0 | 0 | 1(1.6%) | 0 | 1(0.5%) |
| 16CNTZ46 | 0 | 0 | 0 | 1(1.6%) | 0 | 1(0.5%) |
| 16CNTZ47 | 0 | 0 | 0 | 1(1.6%) | 0 | 1(0.5%) |
| **A4** | **40(61.5%)** | **11(50.0%)** | **19(61.3%)** | **45(73.8%)** | **10(66.7%)** | **125(64.4%)** |
| 16CNTZ01 | 26(40.0%) | 9(40.9%) | 10(32.3%) | 25(41.0%) | 8(53.3%) | 78(40.2%) |
| 16CNTZ22 | 4(6.2%) | 1(4.5%) | 4(12.9%) | 9(14.8%) | 1(6.7%) | 19(9.8%) |
| 16CNTZ15 | 3(4.6%) | 0 | 2(6.5%) | 1(1.6%) | 0 | 6(3.1%) |
| 16CNTZ19 | 2(3.1%) | 1(4.5%) | 0 | 2(3.3%) | 0 | 5(2.6%) |
| 16CNTZ06 | 1(1.5%) | 0 | 0 | 1(1.6%) | 0 | 2(1.0%) |
| 16CNTZ17 | 2(3.1%) | 0 | 0 | 0 | 0 | 2(1.0%) |
| 16CNTZ02 | 0 | 0 | 0 | 1(1.6%) | 0 | 1(0.5%) |
| 16CNTZ03 | 0 | 0 | 0 | 1(1.6%) | 0 | 1(0.5%) |
| 16CNTZ05 | 1(1.5%) | 0 | 0 | 0 | 0 | 1(0.5%) |
| 16CNTZ09 | 0 | 0 | 0 | 1(1.6%) | 0 | 1(0.5%) |
| 16CNTZ10 | 0 | 0 | 0 | 0 | 1(6.7%) | 1(0.5%) |
| 16CNTZ11 | 0 | 0 | 1(3.2%) | 0 | 0 | 1(0.5%) |
| 16CNTZ12 | 1(1.5%) | 0 | 0 | 0 | 0 | 1(0.5%) |
| 16CNTZ14 | 0 | 0 | 0 | 1(1.6%) | 0 | 1(0.5%) |
| 16CNTZ16 | 0 | 0 | 1(3.2%) | 0 | 0 | 1(0.5%) |
| 16CNTZ20 | 0 | 0 | 0 | 1(1.6%) | 0 | 1(0.5%) |
| 16CNTZ24 | 0 | 0 | 0 | 1(1.6%) | 0 | 1(0.5%) |
| 16CNTZ25 | 0 | 0 | 0 | 1(1.6%) | 0 | 1(0.5%) |
| 16CNTZ26 | 0 | 0 | 1(3.2%) | 0 | 0 | 1(0.5%) |
| **A5^*^** | **7(10.8%)** | **0** | **1(3.2%)** | **2(3.3%)** | **2(13.3%)** | **12(6.2%)** |
| 16CNTZ59 | 6(9.2%) | 0 | 1(3.2%) | 1(1.6%) | 1(6.7%) | 9(4.6%) |
| 16CNTZ56 | 0 | 0 | 0 | 0 | 1(6.7%) | 1(0.5%) |
| 16CNTZ57 | 1(1.5%) | 0 | 0 | 0 | 0 | 1(0.5%) |
| 16CNTZ60 | 0 | 0 | 0 | 1(1.6%) | 0 | 1(0.5%) |
| **non-A** | **1(1.5%)** | **1(4.5%)** | **2(6.5%)** | **1(1.6%)** | **0** | **5(2.6%)** |
| 16CNTZ64 | 0 | 0 | 1(3.2%) | 1(1.6%) | 0 | 2(1.0%) |
| 16CNTZ61 | 1(1.5%) | 0 | 0 | 0 | 0 | 1(0.5%) |
| 16CNTZ62 | 0 | 0 | 1(3.2%) | 0 | 0 | 1(0.5%) |
| 16CNTZ63 | 0 | 1(4.5%) | 0 | 0 | 0 | 1(0.5%) |
| **Total** | **65** | **22** | **31** | **61** | **15** | **194** |
| **HPV31** |  |  |  |  |  |  |
| **A2** | **20(48.8%)** | **10(66.7%)** | **12(70.6%)** | **7(70.0%)** | **0** | **49(59.0%)** |
| 31CNTZ07 | 10(24.4%) | 4(26.7%) | 8(47.1%) | 5(50.0%) | 0 | 27(32.5%) |
| 31CNTZ04 | 7(17.1%) | 3(20.0%) | 3(17.6%) | 1(10.0%) | 0 | 14(16.9%) |
| 31CNTZ02 | 1(2.4%) | 2(13.3%) | 0 | 0 | 0 | 3(3.6%) |
| 31CNTZ01 | 2(4.9%) | 0 | 0 | 0 | 0 | 2(2.4%) |
| 31CNTZ05 | 0 | 0 | 0 | 1(10.0%) | 0 | 1(1.2%) |
| 31CNTZ06 | 0 | 1(6.7%) | 0 | 0 | 0 | 1(1.2%) |
| 31CNTZ10 | 0 | 0 | 1(5.9%) | 0 | 0 | 1(1.2%) |
| **B1** | **2(4.9%)** | **1(6.7%)** | **0** | **0** | **0** | **3(3.6%)** |
| 31CNTZ12 | 2(4.9%) | 1(6.7%) | 0 | 0 | 0 | 3(3.6%) |
| **C** | **19(46.3%)** | **4(26.7%)** | **5(29.4%)** | **3(30.0%)** | **0** | **31(37.3%)** |
| 31CNTZ15 | 11(26.8%) | 4(26.7%) | 5(29.4%) | 1(10.0%) | 0 | 21(25.3%) |
| 31CNTZ13 | 7(17.1%) | 0 | 0 | 1(10.0%) | 0 | 8(9.6%) |
| 31CNTZ14 | 1(2.4%) | 0 | 0 | 0 | 0 | 1(1.2%) |
| 31CNTZ16 | 0 | 0 | 0 | 1(10.0%) | 0 | 1(1.2%) |
| **Total** | **41** | **15** | **17** | **10** | **0** | **83** |
| **HPV33**^[22]^ |  |  |  |  |  |  |
| **A1** | **39(68.4%)** | **13(76.5%)** | **12(75.0%)** | **17(85.0)** | **0** | **81(73.6%)** |
| 33CNTZ01 | 36(63.2%) | 12(70.6%) | 11(68.8%) | 15(75.0%) | 0 | 74(67.3%) |
| 33CNTZ02 | 0 | 1(5.9%) | 1(6.3%) | 1(5.0%) | 0 | 3(2.7%) |
| 33CNTZ05 | 1(1.8%) | 0 | 0 | 0 | 0 | 1(0.9%) |
| 33CNTZ08 | 1(1.8%) | 0 | 0 | 1(5.0%) | 0 | 2(1.8%) |
| 33CNTZ09 | 1(1.8%) | 0 | 0 | 0 | 0 | 1(0.9%) |
| **A2** | **0** | **0** | **1(6.3%)** | **0** | **0** | **1(0.9%)** |
| 33CNTZ10 | 0 | 0 | 1(6.3%) | 0 | 0 | 1(0.9%) |
| **A3** | **18(31.6%)** | **4(23.5%)** | **3(18.8%)** | **3(15.0%)** | **0** | **28(25.5%)** |
| 33CNTZ11 | 13(22.8%) | 3(17.6%) | 3(18.8%) | 3(15.0%) | 0 | 22(20.0%) |
| 33CNTZ12 | 1(1.8%) | 1(5.9%) | 0 | 0 | 0 | 2(1.8%) |
| 33CNTZ13 | 2(3.5%) | 0 | 0 | 0 | 0 | 2(1.8%) |
| 33CNTZ14 | 1(1.8%) | 0 | 0 | 0 | 0 | 1(0.9%) |
| 33CNTZ15 | 1(1.8%) | 0 | 0 | 0 | 0 | 1(1.8%) |
| **Total** | **57** | **17** | **16** | **20** | **0** | **110** |
| **HPV35** |  |  |  |  |  |  |
| **A1** | **56(98.2%)** | **8(100%)** | **8(100%)** | **4(100%)** | **0** | **76(98.7%)** |
| 35CNTZ01 | 34(59.6%) | 4(50.0%) | 6(75.0%) | 3(75.0%) | 0 | 47(61.0%) |
| 35CNTZ03 | 21(36.8%) | 2(25.0%) | 2(25.0%) | 1(25.0%) | 0 | 26(33.8%) |
| 35CNTZ04 | 0 | 2(25.0%) | 0 | 0 | 0 | 2(2.6%) |
| 35CNTZ02 | 1(1.8%) | 0 | 0 | 0 | 0 | 1(1.3%) |
| **A2** | **1(1.8%)** | **0** | **0** | **0** | **0** | **1(1.3%)** |
| 35CNTZ05 | 1(1.8%) | 0 | 0 | 0 | 0 | 1(1.3%) |
| **Total** | **57** | **8** | **8** | **4** | **0** | **77** |
| **HPV52**^[23]^ |  |  |  |  |  |  |
| **A1** | **2(3.0%)** | **1(1.9%)** | **1(2.3%)** | **0** | **0** | **4(2.1%)** |
| 52CNTZ27 | 2(3.0%) | 1(1.9%) | 1(2.3%) | 0 | 0 | 4(2.1%) |
| **B** | **64(95.5%)** | **52(96.3%)** | **42(95.5%)** | **23(95.8%)** | **0** | **181(95.8%)** |
| 52CNTZ05 | 39(58.2%) | 31(57.4%) | 26(59.1%) | 12(50.0%) | 0 | 108(57.1%) |
| 52CNTZ12 | 16(23.9%) | 15(27.8%) | 8(18.2%) | 8(33.3%) | 0 | 47(24.9%) |
| 52CNTZ17 | 2(3.0%) | 2(3.7%) | 2(4.5%) | 0 | 0 | 6(3.2%) |
| 52CNTZ21 | 2(3.0%) | 0 | 3(6.8%) | 0 | 0 | 5(2.6%) |
| 52CNTZ13 | 0 | 1(1.9%) | 0 | 1(4.2%) | 0 | 2(1.1%) |
| 52CNTZ23 | 0 | 1(1.9%) | 1(2.3%) | 0 | 0 | 2(1.1%) |
| 52CNTZ02 | 0 | 1(1.9%) | 0 | 0 | 0 | 1(0.5%) |
| 52CNTZ04 | 0 | 0 | 1(2.3%) | 0 | 0 | 1(0.5%) |
| 52CNTZ07 | 1(1.5%) | 0 | 0 | 0 | 0 | 1(0.5%) |
| 52CNTZ08 | 1(1.5%) | 0 | 0 | 0 | 0 | 1(0.5%) |
| 52CNTZ09 | 1(1.5%) | 0 | 0 | 0 | 0 | 1(0.5%) |
| 52CNTZ10 | 0 | 1(1.9%) | 0 | 0 | 0 | 1(0.5%) |
| 52CNTZ11 | 0 | 0 | 0 | 1(4.2%) | 0 | 1(0.5%) |
| 52CNTZ14 | 0 | 0 | 0 | 1(4.2%) | 0 | 1(0.5%) |
| 52CNTZ16 | 1(1.5%) | 0 | 0 | 0 | 0 | 1(0.5%) |
| 52CNTZ18 | 0 | 0 | 1(2.3%) | 0 | 0 | 1(0.5%) |
| 52CNTZ19 | 1(1.5%) | 0 | 0 | 0 | 0 | 1(0.5%) |
| **C2** | **1(1.5%)** | **0** | **2(4.5%)** | **1(4.2%)** | **0** | **4(2.1%)** |
| 52CNTZ25 | 1(1.5%) | 0 | 1(2.3%) | 0 | 0 | 2(1.1%) |
| 52CNTZ26 | 0 | 0 | 1(2.3%) | 1(4.2%) | 0 | 2(1.1%) |
| **Total** | **67** | **53** | **45** | **24** | **0** | **189** |
| **HPV58**^[24]^ |  |  |  |  |  |  |
| **A1** | **36(75.0%)** | **10(66.7%)** | **17(70.8%)** | **13(65.0%)** | **0** | **76(71.0%)** |
| 58CNTZ10 | 11(22.9%) | 4(26.7%) | 3(12.5%) | 6(30.0%) | 0 | 24(22.4%) |
| 58CNTZ01 | 11(22.9%) | 4(26.7%) | 3(12.5%) | 4(20.0%) | 0 | 22(20.6%) |
| 58CNTZ02 | 7(14.6%) | 1(6.7%) | 9(37.5%) | 1(5.0%) | 0 | 18(16.8%) |
| 58CNTZ04 | 1(2.1%) | 0 | 0 | 1(5.0%) | 0 | 2(1.9%) |
| 58CNTZ03 | 0 | 0 | 1(4.2%) | 0 | 0 | 1(0.9%) |
| 58CNTZ06 | 1(2.1%) | 0 | 0 | 0 | 0 | 1(0.9%) |
| 58CNTZ07 | 1(2.1%) | 0 | 0 | 0 | 0 | 1(0.9%) |
| 58CNTZ08 | 1(2.1%) | 0 | 0 | 0 | 0 | 1(0.9%) |
| 58CNTZ09 | 1(2.1%) | 0 | 0 | 0 | 0 | 1(0.9%) |
| 58CNTZ11 | 1(2.1%) | 0 | 0 | 0 | 0 | 1(0.9%) |
| 58CNTZ12 | 0 | 1(6.7%) | 0 | 0 | 0 | 1(0.9%) |
| 58CNTZ13 | 1(2.1%) | 0 | 0 | 0 | 0 | 1(0.9%) |
| 58CNTZ15 | 0 | 0 | 1(4.2%) | 1(5.0%) | 0 | 2(1.9%) |
| **A2** | **9(18.8%)** | **3(20.0%)** | **3(12.5%)** | **2(10.0%)** | **0** | **17(15.9%)** |
| 58CNTZ17 | 8(16.7%) | 3(20.0%) | 2(8.3%) | 2(10.0%) | 0 | 15(14.0%) |
| 58CNTZ20 | 0 | 0 | 1(4.2%) | 0 | 0 | 1(0.9%) |
| 58CNTZ22 | 1(2.1%) | 0 | 0 | 0 | 0 | 1(0.9%) |
| **A3** | **3(6.3%)** | **2(13.3)** | **4(16.7%)** | **5(25.0%)** | **0** | **14(13.1%)** |
| 58CNTZ23 | 3(6.3%) | 2(13.3%) | 3(12.5%) | 5(25.0%) | 0 | 13(12.1%) |
| 58CNTZ25 | 0 | 0 | 1(4.2%) | 0 | 0 | 1(0.9%) |
| **Total** | **48** | **15** | **24** | **20** | **0** | **107** |
| CIN: cervical intraepithelial neoplasia  Some of the oncogenic risk association data for α-9 HPV types have been published in our previous studies, including HPV16^[21]^, 33^[22]^, 52^[23]^ and 58^[24]^.  **^*^** Notably, the HPV16 A5 variant in our study^[21]^ is not in the same cluster as the A5 variant reported in recent studies in Japan (Hirose et al. Viruses.2019,11(4):350; Hashida et al. Cancer Sci. 2021,112(10):4404; Tanaka et al. Viruses. 2022,14(3):464), which share a common ancestor with the Thai isolate FJ610151 (Lurchachaiwong et al. Virus Genes. 2009, 39(1):30). In this study, the particular nonsynonymous substitution in HPV16 E7 is C790T (R77L), while it is E7 A645C (L28F) in Japan. | | | | | | |
