# Supplementary figures and images for: Phylogenetic analysis and antigenic epitope prediction for E6 and E7 of Alpha-papillomavirus 9 in Taizhou, China
Source: BMC Genomics. 2024 May 22;25:507. doi: 10.1186/s12864-024-10411-1 (PMC11110188; doi:10.1186/s12864-024-10411-1)

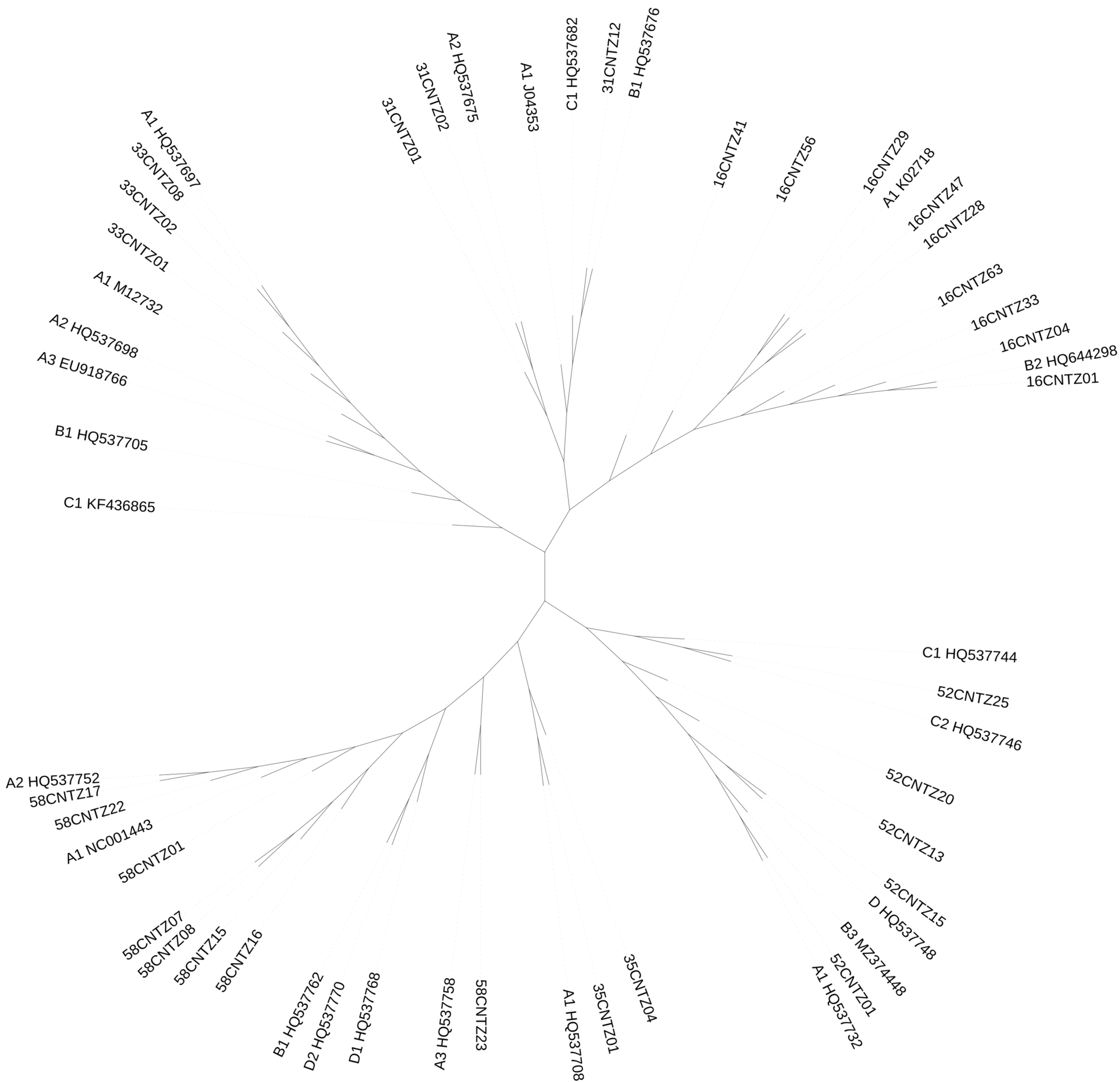

Figure S2. Phylogenetic tree of -9 HPV variants based on E7 amino acid sequence

Supplement: Supplementary file 5 — Supplementary Material 5. [file 12864_2024_10411_MOESM5_ESM.pdf]
