# Supplementary material for: Phylogenetic analysis and antigenic epitope prediction for E6 and E7 of Alpha-papillomavirus 9 in Taizhou, China
Source: BMC Genomics. 2024 May 22;25:507. doi: 10.1186/s12864-024-10411-1 (PMC11110188; doi:10.1186/s12864-024-10411-1)
Supplement: Supplementary file 6 — Supplementary Material 6. [file 12864_2024_10411_MOESM6_ESM.pdf]

```
              ....|....| ....|....| ....|....| ....|....| ....|....| ....|....|
                     10         20         30         40         50         60
PDB|7UAJ|B    -------MFQ DPQERPRKLP QLCTELQTTI HDIILECVYC KQQLLRREVY DFAFRDLCIV
16CNTZ01 E6   MHQKRTAMFQ DPQERPRKLP QLCTELQTTI HEIILECVYC KQQLLRREVY DFAFRDLCIV
PDB|6SLM|A    MFKNPAERPR KLHELSSALE IPYDELRLNC VYCKGQLTET EVLDFAFTDL TIVYRDDTPH
31CNTZ04 E6   MFKNPAERPR KLHELSSALE IPYDELRLNC VYCKGQLTET EVLDFAFTDL TIVYRDDTPH
PDB|4XR8|C    MFQDPQERPR KLPQLCTELQ TTIHDIILEC VYCKQQLLRR EVYDFAFRDL CIVYRDGNPY
33CNTZ01 E6   MFQDTEEKPR TLHDLCQALE TTIHNIELQC VECKKPLQRS EVYDFAFADL TVVYREGNPF
PDB|4XR8|C    MFQDPQERPR KLPQLCTELQ TTIHDIILEC VYCKQQLLRR EVYDFAFRDL CIVYRDGNPY
35CNTZ01 E6   MFQDPAERPY KLHDLCNEVE ESIHEICLNC VYCKQELQRS EVYDFACYDL CIVYREGQPY
PDB|4XR8|C    MFQDPQERPR KLPQLCTELQ TTIHDIILEC VYCKQQLLRR EVYDFAFRDL CIVYRDGNPY
52CNTZ05 E6   MFEDPATRPR TLHELCEVLE ESVHEIRLQC VQCKKELQRR EVYKFLFTDL RIVYRDNNPY
PDB|4XR8|C    MFQDPQERPR KLPQLCTELQ TTIHDIILEC VYCKQQLLRR EVYDFAFRDL CIVYRDGNPY
58CNTZ01 E6   MFQDAEEKPR TLHDLCQALE TSVHEIELKC VECKKTLQRS EVYDFVFADL RIVYRDGNPF

              ....|....| ....|....| ....|....| ....|....| ....|....| ....|....|
                     70         80         90        100        110        120
PDB|7UAJ|B    YRDGNPYAVC DKCLKFYSKI SEYRHYCYSL YGTTLEQQYN KPLSDLLIRC INCQKPLSPE
16CNTZ01 E6   YRDGNPYAVC DKCLKFYSKI SEYRHYCYSL YGTTLEQQYN KPLCDLLIRC INCQKPLCPE
PDB|6SLM|A    GVCTKCLRFY SKVSEFRWYR YSVYGTTLEK LTNKGISDLL IRCITCQRPL SPEEKQRHLD
31CNTZ04 E6   GVCTKCLRFY SKVSEFRWYR YSVYGTTLEK LTNKGICDLL IRCITCQRPL CPEEKQRHLD
PDB|4XR8|C    AVCDKCLKFY SKISEYRHYS YSLYGTTLEQ QYNKPLSDLL IRCINCQKPL SPEEKQRHLD
33CNTZ01 E6   GICKLCLRFL SKISEYRHYN YSVYGNTLEQ TVKKPLNEIL IRCIICQRPL CPQEKKRHVD
PDB|4XR8|C    AVCDKCLKFY SKISEYRHYS YSLYGTTLEQ QYNKPLSDLL IRCINCQKPL SPEEKQRHLD
35CNTZ01 E6   GVCMKCLKFY SKISEYRRYR YSVYGETLEK QCNKQLCHLL IRCITCQKPL CPVEKQRHLE
PDB|4XR8|C    AVCDKCLKFY SKISEYRHYS YSLYGTTLEQ QYNKPLSDLL IRCINCQKPL SPEEKQRHLD
52CNTZ05 E6   GVCIMCLRFL SKISEYRHYQ YSLYGKTLEE RVRKPLSEIT IRCIICQTPL CPEEKERHVN
PDB|4XR8|C    AVCDKCLKFY SKISEYRHYS YSLYGTTLEQ QYNKPLSDLL IRCINCQKPL SPEEKQRHLD
58CNTZ01 E6   AVCKVCLRLL SKISEYRHYN YSLYGDTLEQ TLKKCLNEIL IRCIICQRPL CPQEKKRHVD

              ....|....| ....|....| ....|....| ....|...
                    130        140        150
PDB|7UAJ|B    EKQRHLDKKQ RFHNIRGRWT GRCMSCSRSS RTRRETQL
16CNTZ01 E6   EKQRHLDKKQ RFHNIRGRWT GRCMSCCRSS RTRRETQL
PDB|6SLM|A    KKKRFHNIGG RWTGRCIACW RRPRTETQV
31CNTZ04 E6   KKKRFHNIGG RWTGRCIACW RRPRTETQV
PDB|4XR8|C    KKQRFHNIRG RWTGRCMSCS RSSRTRR--
33CNTZ01 E6   LNKRFHNISG RWAGRCAACW RSRRRETAL
PDB|4XR8|C    KKQRFHNIRG RWTGRCMSCS RSSRTRR--
35CNTZ01 E6   EKKRFHNIGG RWTGRCMSCW KPTRRETEV
PDB|4XR8|C    KKQRFHNIRG RWTGRCMSCS RSSRTRR-
52CNTZ05 E6   ANKRFHNIMG RWTGRCSECW RPRPVTQV
PDB|4XR8|C    KKQRFHNIRG RWTGRCMSCS RSSRTRR--
58CNTZ01 E6   LNKRFHNISG RWTGRCAVCW RPRRRQTQV
```

Figure S3. The template-target pairwise sequence alignment for α-9 HPV E6
