# Supplementary material for: Phylogenetic analysis and antigenic epitope prediction for E6 and E7 of Alpha-papillomavirus 9 in Taizhou, China
Source: BMC Genomics. 2024 May 22;25:507. doi: 10.1186/s12864-024-10411-1 (PMC11110188; doi:10.1186/s12864-024-10411-1)
Supplement: Supplementary file 7 — Supplementary Material 7. [file 12864_2024_10411_MOESM7_ESM.pdf]

```
                ....|....| ....|....| ....|....| ....|....| ....|....| ....|....|
                         10         20         30         40         50         60
PDB|2B9D|A      ---------- ---------- ---------- ---------- ---------Q PYAVVASCAY
16CNTZ01 E7     MHGDTPTLHE YMLDLQPETT DLYCYEQLSD SSEEEDEIDG PAGQAEPDRA HYNIVTFCCK
PDB|2B9D|A      ---------- ---------- ---------- ---------- ---------Q PYAVVASCAY
31CNTZ04 E7     MRGETPTLQD YVLDLQPEAT DLYCYEQLPD SSDEEDVIDS PAGQAEPDTS NYNIVTFCCQ
PDB|2B9D|A      ---------- ---------- ---------- ---------- ---------Q PYAVVASCAY
33CNTZ01 E7     MRGHKPTLKE YVLDLYPEPT DLYCYEQLSD SSDEDEGLDR PDGQAQPATA DYYIVTCCHT
PDB|2B9D|A      ---------- ---------- ---------- ---------- ---------- QPYAVVASCA
35CNTZ01 E7     MHGEITTLQD YVLDLEPEAT DLYCYEQLCD SSEEEEDTID GPAGQAKPDT SNYNIVTSCC
PDB|2B9D|A      ---------- ---------- ---------- ---------- ---------- QPYAVVASCA
52CNTZ05 E7     MRGDKATIKD YILDLQPETT DLHCYEQLGD SSDEEDTDGV DRPDGQAEQA TSNYYIVTYC
PDB|2B9D|A      ---------- ---------- ---------- ---------- ---------- QPYAVVASCA
58CNTZ01 E7     MRGNNPTLRE YILDLHPEPT DLFCYEQLCD SSDEDEIGLD GPDGQAQPAT ANYYIVTCCY


                ....|....| ....|....| ....|....| ....|....
                         70         80         90
PDB|2B9D|A      CEKLVRLTVL ADHSAIRQLE EMLLRSLNIV CPLCTLQR
16CNTZ01 E7     CDSTLRLCVQ STHVDIRTLE DLLMGTLGIV CPICSQKP
PDB|2B9D|A      CEKLVRLTVL ADHSAIRQLE EMLLRSLNIV CPLCTLQ-
31CNTZ04 E7     CESTLRLCVQ STQVDIRILQ ELLMGSFGIV CPNCSTRL
PDB|2B9D|A      CEKLVRLTVL ADHSAIRQLE EMLLRSLNIV CPLCTLQ
33CNTZ01 E7     CNTTVRLCVN STASDLRTIQ QLLMGTVNIV CPTCAQQ
PDB|2B9D|A      YCEKLVRLTV LADHSAIRQL EEMLLRSLNI VCPLCTLQ-
35CNTZ01 E7     KCEATLRLCV QSTHIDIRKL EDLLMGTFGI VCPGCSQRA
PDB|2B9D|A      YCEKLVRLTV LADHSAIRQL EEMLLRSLNI VCPLCTLQ-
52CNTZ05 E7     HSCDSTLRLC IHSTATDLRT LQQMLLGTLQ VVCPGCARL
PDB|2B9D|A      YCEKLVRLTV LADHSAIRQL EEMLLRSLNI VCPLCTLQ
58CNTZ01 E7     TCGTTVRLCI NSTTTDVRTL QQLLMGTCTI VCPSCAQQ
```

Figure S4. The template-target pairwise sequence alignment for α-9 HPV E7
