# Supplementary material for: Phylogenetic analysis and antigenic epitope prediction for E6 and E7 of Alpha-papillomavirus 9 in Taizhou, China
Source: BMC Genomics. 2024 May 22;25:507. doi: 10.1186/s12864-024-10411-1 (PMC11110188; doi:10.1186/s12864-024-10411-1)
Supplement: Supplementary file 8 — Supplementary Material 8. [file 12864_2024_10411_MOESM8_ESM.pdf]

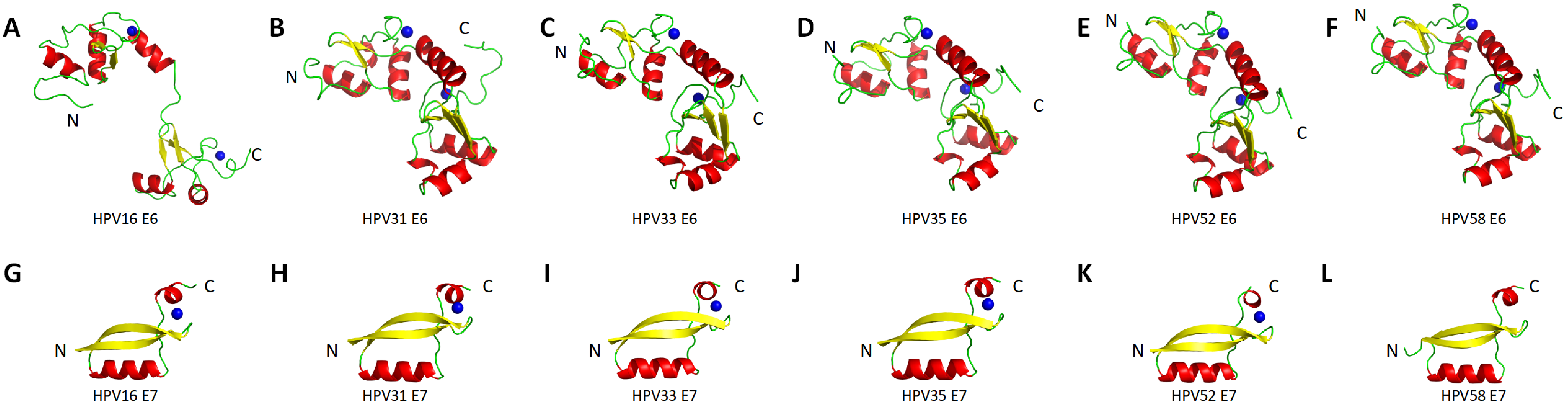

Figure S5. 3D structural models of α-9 HPV E6 or E7 proteins (cartoon representation).
A. HPV16 E6, B. HPV31 E6, C. HPV33 E6, D. HPV35 E6, E. HPV52 E6, F. HPV58 E6, G. HPV16 E7, H. HPV31 E7, I. HPV33 E7, J. HPV35 E7, K. HPV52 E7, L. HPV58 E7.
