# Supplementary material for: Phylogenetic analysis and antigenic epitope prediction for E6 and E7 of Alpha-papillomavirus 9 in Taizhou, China
Source: BMC Genomics. 2024 May 22;25:507. doi: 10.1186/s12864-024-10411-1 (PMC11110188; doi:10.1186/s12864-024-10411-1)
Supplement: Supplementary file 9 — Supplementary Material 9. [file 12864_2024_10411_MOESM9_ESM.pdf]

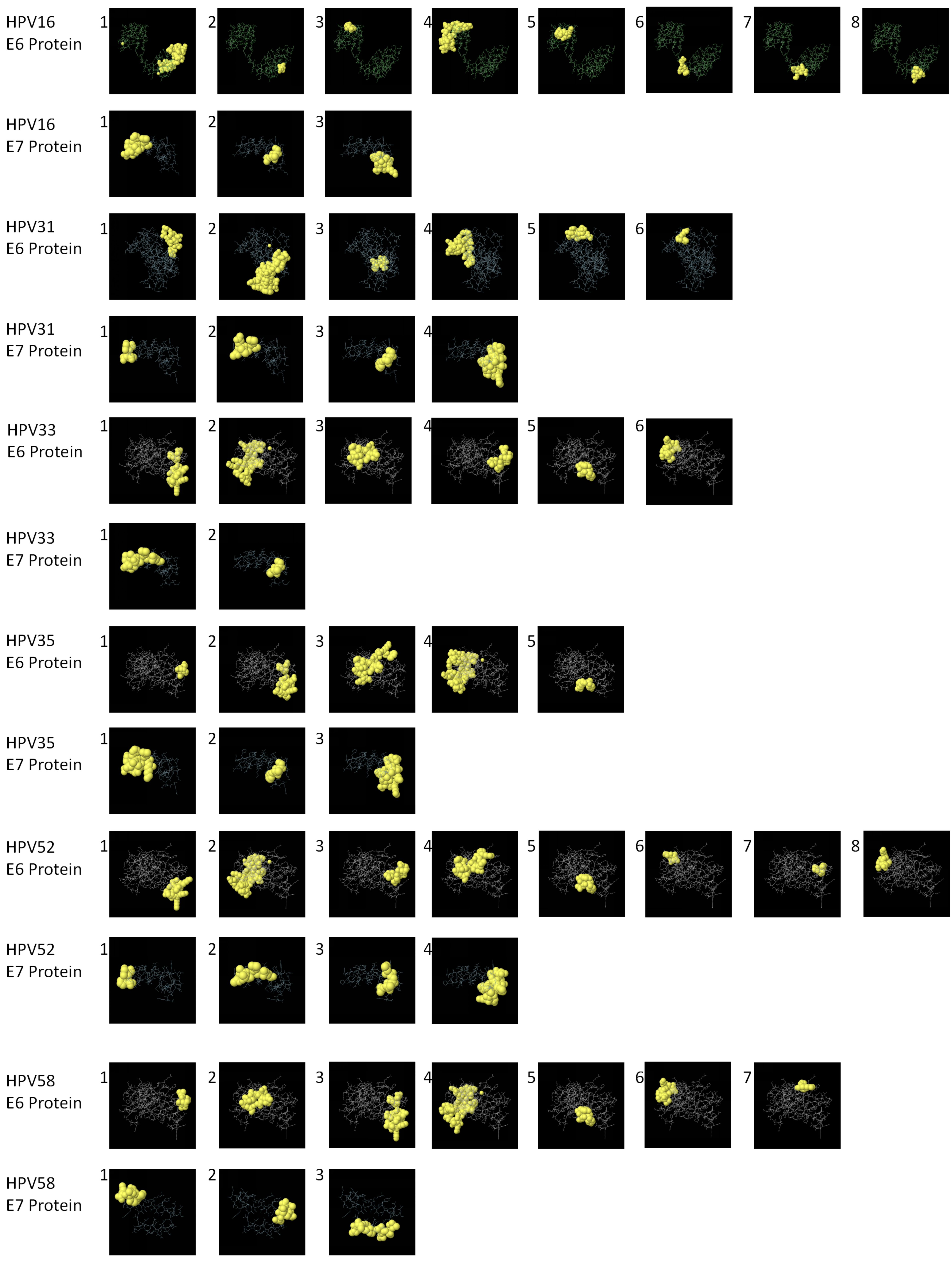

Figure S6. ElliPro predicted conformational B-cell epitopes of E6 and E7 proteins of α-9 genus HPV represented in 3D. The numbers of the predicted epitope regions for each protein correspond to the number of predictions in Table 5. Orientation of the proteins is the same as in Figure 5S.
